# Natriuretic Peptide Receptor B modulates the proliferation of the cardiac cells expressing the Stem Cell Antigen-1

**DOI:** 10.1038/srep41936

**Published:** 2017-02-09

**Authors:** Stéphanie Rignault-Clerc, Christelle Bielmann, Lucas Liaudet, Bernard Waeber, François Feihl, Nathalie Rosenblatt-Velin

**Affiliations:** 1 Unité de Physiopathologie Clinique Centre Hospitalier Universitaire Vaudois and University of Lausanne, Bugnon 7a, 1005 Lausanne, Switzerland; 2 Service de Médecine Intensive Adulte, Centre Hospitalier Universitaire Vaudois and University of Lausanne, Switzerland

## Abstract

Brain Natriuretic Peptide (BNP) injections in adult “healthy” or infarcted mice led to increased number of non-myocyte cells (NMCs) expressing the nuclear transcription factor Nkx2.5. The aim of this study was to identify the nature of the cells able to respond to BNP as well as the signaling pathway involved. BNP treatment of neonatal mouse NMCs stimulated Sca-1^+^ cell proliferation. The Sca-1^+^ cells were characterized as being a mixed cell population involving fibroblasts and multipotent precursor cells. Thus, BNP treatment led also to increased number of Sca-1^+^ cells expressing Nkx2.5, in Sca-1^+^ cell cultures *in vitro* and *in vivo*, in the hearts of neonatal and adult infarcted mice. Whereas BNP induced Sca-1^+^ cell proliferation via NPR-B receptor and protein kinase G activation, CNP stimulated Sca-1^+^ cell proliferation via NPR-B and a PKG-independent mechanism. We highlighted here a new role for the natriuretic peptide receptor B which was identified as a target able to modulate the proliferation of the Sca-1^+^ cells. The involvement of NPR-B signaling in heart regeneration has, however, to be further investigated.

As the endogenous cardiac precursor cells (CPCs) have been shown to contribute to heart regeneration in physiological as well as in pathophysiological conditions^1^,^2^,^3^,^4^, the challenge in the coming years is to increase their potential to proliferate and differentiate into mature functional cardiomyocytes.

One of the major problems which limited the development of strategies aimed to improve heart regeneration, is the identification of the CPCs which remains a difficult task, due to the lack of a defined, highly specific marker. These last years, the use of different markers (most notably the c-kit and the Stem Cell antigen-1 (Sca-1) proteins and the islet-1 nuclear transcription factor) as well as different isolation methods (colony forming assays, dye-efflux methods, flow cytometry cell sorting) generated confusing results^3^,^4^,^5^,^6^,^7^,^8^,^9^. The fact that the proteins used to identify the CPCs are also expressed on cardiac and non-cardiac “differentiated” cells made this identification even more difficult. Thus, c-kit and Sca-1 proteins have been identified on endothelial cells and on fibroblasts. Furthermore, cardiac fibroblasts expressing Sca-1 for 79% of them, express also a high number of cardiogenic transcription factors, such as Hand2, Tbx20, Tbx5 and Nkx2.5, which further complicate the story^10^.

An explanation of all these controversies could reside in the heterogeneity of the c-kit^+^ or Sca-1^+^ cell population isolated from neonatal or adult hearts. Thus Hatzistergos *et al*. identified in the heart two subsets of c-kit^+^ cells: one originating from the cardiac neural crest, expressing Nkx2.5 and able to differentiate into cardiomyocytes and one of mesodermal origin which contributes rather to endothelial cells^11^,^12^,^13^. Among the Sca-1^+^ side population isolated from adult murine hearts only a subset of cells expressing the platelet-derived growth factor receptor-α (PDGFRα) is considered as clonogenic^14^. Thus, to discriminate between CPCs and differentiated cells expressing the c-kit or Sca-1 protein, the molecular and cellular analysis is insufficient and the origin of the cells as well as their plasticity have also to be taken in count.

The lack of knowledge concerning the mechanisms controlling the proliferation and differentiation of CPCs *in vivo* is another limiting factor in the field of heart regeneration. Although more and more results demonstrate the involvement of “paracrine signals”, their identification as well as their origin are not yet known. Interestingly, in senescent hearts, the proliferation of c-kit^+^ cells can be re-activated by the stem cell factor^15^. Bone Morphogenetic Protein (BMP) gradient in the heart seems also to modulate the differentiation of the c-kit^+^ cells of cardiac neural crest origin^12^. Using R26R-confetti mice, it was shown that Sca-1^+^ cells contribute more to cardiomyocyte renewal in physiological (i.e. during physiological growth and ageing) than in pathophysiological (i.e. after ischemia or pressure overload) conditions^4^. Thus, the relative “non-activation” of the Sca-1^+^ CPCs in the ischemic hearts could be due either to the presence of an “inactivating” factor or to the absence of a “stimulating” factor. Identifying the factors able to stimulate CPC proliferation and differentiation will be essential for further development of therapeutically strategies aimed to stimulate heart regeneration even in elderly patients suffering from cardiac vascular diseases.

Recently, we identified a factor able to increase the number of newly formed cardiomyocytes in mouse hearts during physiological growth and after myocardial infarction (MI)^16^. The Brain Natriuretic Peptide (BNP) is a cardiac hormone secreted through a constitutive mechanism by ventricular cardiomyocytes, fibroblasts, endothelial cells and even by infiltrating neutrophils, T-cells and macrophages after MI^17^. Interestingly, BNP is also secreted by immature cells, such as embryonic stem cells^18^, satellite cells^19^ or CPCs^20^. BNP binds to two guanylyl cyclase receptors, denoted NPR-A and NPR-B, which leads to the generation of intracellular cGMP^21^. The accumulation of cGMP in the cytoplasm activates protein kinase G (PKG) and the phosphodiesterases 2, 3 or 5^21^.

We recently demonstrated that BNP injections into neonatal and adult healthy or infarcted mice led to reduced heart dilation associated at the cellular level to increased number of Nkx2.5^+^ α actinin^−^ cells and newly formed cardiomyocytes^16^. *In vitro,* BNP clearly stimulated the proliferation of the Nkx2.5^+^ non myocyte cells (NMCs) and their differentiation into cardiomyocytes. Thus, in this report we determined the nature of the cell subset (i.e. from c-kit or Sca-1 origin) responding to BNP stimulation among NMCs and we identified the signaling pathway involved.

## Results

### BNP increases the number of Sca-1^+^ cells

**Figure 1:**
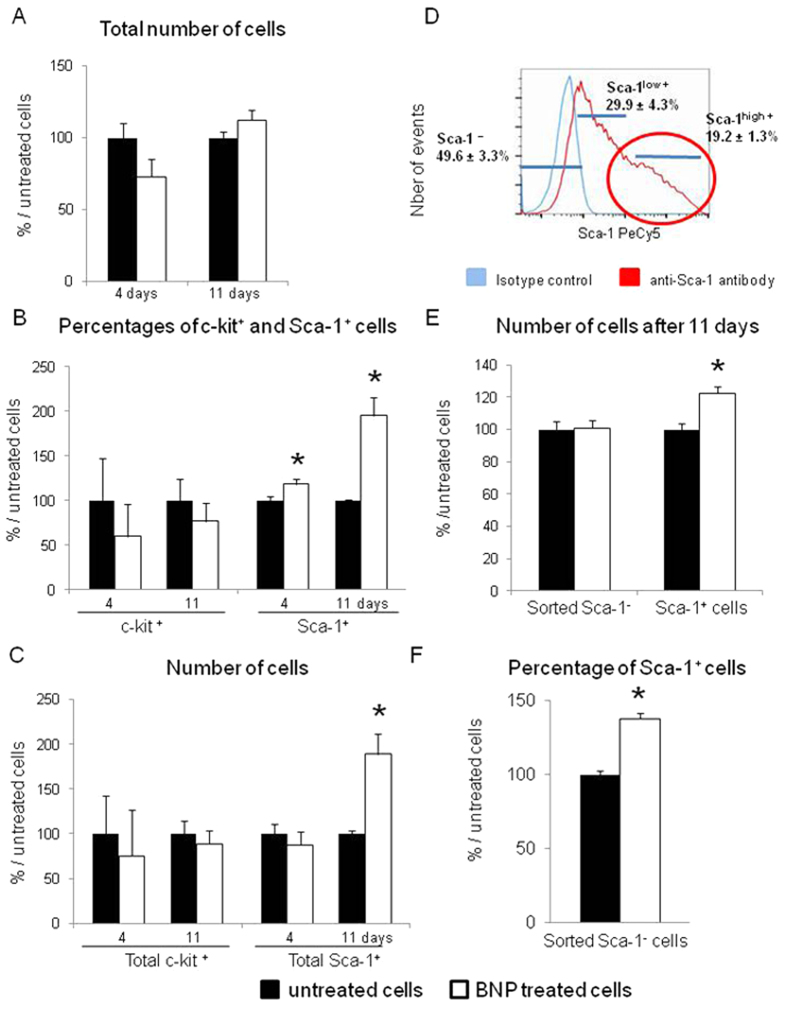
BNP stimulates Sca-1^+^ cell proliferation. (**A**) Non myocyte cells (NMCs) were isolated from neonatal hearts of C57BL/6 mice, cultured 4 and 11 days with or without BNP (untreated cells) and counted. (**B**) Percentages of c-kit^+^ and Sca-1^+^ cells obtained by flow cytometry analysis on BNP treated or untreated NMCs. (**C**) Number of cells expressing the c-kit or the Sca-1 protein in NMCs treated or not with BNP for 4 and 11 days calculated with the total number of cells and the percentages of the c-kit^+^ and Sca-1^+^ cells. (**A**–**C**) n = 8 and 16 different experiments after 4 and 11 days of culture, respectively. (**D**) Representative histogram of NMC sorting for Sca-1 expression. The numbers represent the percentage of the cells compared to the total number of sorted NMCs. n = 18–43 different experiments. (**E**) Number of sorted Sca-1^−^ and Sca-1^high+^ cells treated or not with BNP for 11 days. n = 6 and 12 for Sca-1^−^ and Sca-1^high+^ cells, respectively. (**F**) Percentages of Sca-1^+^ cells among sorted Sca-1^−^ cells treated or not with BNP for 9 days. n = 4 different experiments. (**A**–**E**) All results expressed as fold-increase above the results obtained in untreated cells. All the results are means ± SEM. *p < 0.05.

To determine whether BNP treatment modified the number of c-kit^+^ or/and Sca-1^+^ cells, flow cytometry analysis using antibodies against c-kit or Sca-1 proteins were performed on NMCs isolated from neonatal mouse hearts and cultured with or without BNP for up to 11 days (i.e. until reaching confluence). BNP treatment didn’t statistically modify the total number of cells (−27%, p = 0.14 at 4 days and +12%, p = 0.12 at 11 days) (Fig. 1A) but increased the percentages of Sca-1 positive cells after 4 (+18%, p = 0.03) and 11 days (+95%, p = 0.0001) (Fig. 1B). The percentages of c-kit^+^ cells remained similar between BNP treated and untreated cells (Fig. 1B). As a consequence, the total number of Sca-1^+^ cells was increased after 11 days of treatment (+89% compared to untreated cells, p = 0.0001) and the number of c-kit^+^ cells remained unchanged (Fig. 1C). Accordingly, mRNA levels coding for Sca-1 was increased in BNP treated cells compared to the untreated ones (Supplemental Fig. 1A).

To determine whether BNP stimulated directly the proliferation of the Sca-1^+^ cells and/or induced the expression of Sca-1 on the Sca-1^−^ cells, cell sorting based on Sca-1 expression was performed on neonatal NMCs (Fig. 1D). Sca-1^−^ and Sca-1^high+^ cells were cultured with or without BNP. After 11 days, BNP treatment increased only the number of Sca-1^+^ cells (+24.5%, p = 0.0006) (Fig. 1E). Immunostainings using antibodies against Sca-1 and the Proliferating Cell Nuclear Antigen (PCNA) allowed to identify proliferating Sca-1^+^ cells (i.e. Sca-1^+^ PCNA^+^ cells) in BNP treated cell culture (Supplemental Fig. 1B). On sorted Sca-1^−^ cells, flow cytometry analysis were performed in order to determine whether BNP treatment induced the expression of the Sca-1 protein (Fig. 1F). Immediately after sorting, no Sca-1^+^ cell among the Sca-1^−^ cells was detected. However, after 9 days of culture, 19% ± 1% of the sorted Sca-1^−^ cells expressed spontaneously the Sca-1 protein. In presence of BNP, the number of Sca-1^+^ cells among sorted Sca-1^−^ cells reached 28% ± 1% (+48% versus untreated NMCs, p < 0.001), demonstrating that BNP stimulated Sca-1 expression on Sca-1^−^ cells.

Thus, increased number of Sca-1^+^ cells among BNP treated NMCs is the consequence of increased Sca-1^+^ cell proliferation and increased expression of the Sca-1 protein on Sca-1^−^ cells.

### Sca-1^+^ cells express NPR-A and NPR-B receptors in neonatal and adult hearts

If we assume that BNP is able to modulate Sca-1^+^ cell proliferation, BNP receptors should be expressed on the Sca-1^+^ cells. We determined by flow cytometry analysis that around 2% of the NMCs isolated from adult or neonatal hearts co-expressed Sca-1 and NPR-A and 4% Sca-1 and NPR-B (Supplemental Table S1). Our results highlighted the presence of three subsets of Sca-1^+^ cells concerning BNP receptor expression in the neonatal and adult hearts: (1) Sca-1^+^ NPR-A^−^ NPR-B^−^ cells, (2) Sca-1^+^ NPR-A^+^ cells expressing or not NPR-B receptor, (3) Sca-1^+^ NPR-A^−^ NPR-B^+^ cells.

The presence in neonatal and adult mouse hearts of Sca-1^+^ NPR-A^+^ and Sca-1^+^ NPR-B^+^ cells was also highlighted by immunostainings (Supplemental Fig. 2, orange and white arrows). Interestingly, some of the Sca-1^+^ NPR-A^+^ and Sca-1^+^ NPR-B^+^ cells co-expressed the nuclear transcription factor Nkx2.5 (white arrows) in neonatal and adult hearts. In neonatal hearts, Sca-1^+^ Nkx2.5^+^ NPR-B^+^ cells were always detected in clusters next to the epicardium (Supplemental Fig. 2A). In adult hearts, Sca-1^+^ Nkx2.5^+^ NPR-B^+^ or NPR-A^+^ cells were located in some “niches” (Supplemental Fig. 2B). In neonatal and adult hearts we detected also Sca-1^−^ Nkx2.5^+^ NPR-B^+^ cells (red arrows). The Sca-1^+^ NPR-A^+^ and Sca-1^+^ NPR-B^+^ cells were detected in adult hearts in smaller numbers than in neonatal hearts. Thus, we performed the next *in vitro* experiments only with neonatal cells.

### BNP treatment increases the number of Sca-1^+^ Nkx2.5^+^ cells *in vitro* and *in vivo*

**Figure 2:**
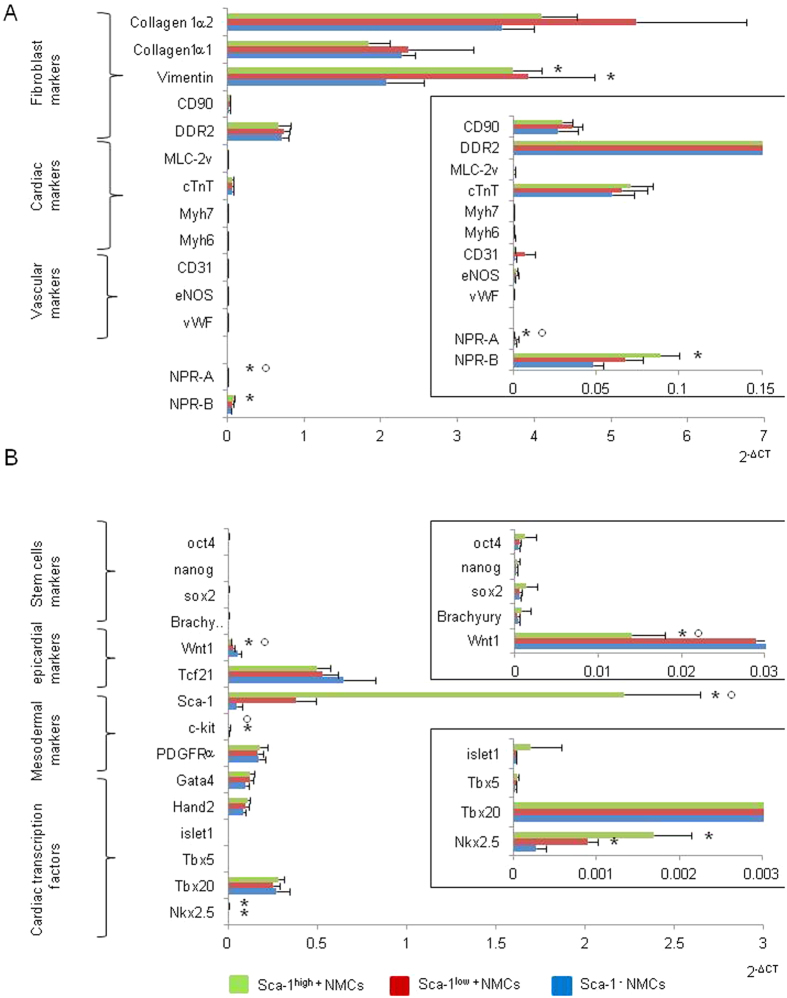
Molecular characterization of sorted Sca-1^−^, Sca-1^low+^ and Sca-1^high+^ cells directly after cell sorting. Quantitative polymerase chain reactions were performed for genes expressed in differentiated cells (**A**) or in undifferentiated cells (**B**). Densities of expression were represented as 2^−ΔCT^ and results are means ± SEM of 6 different cell sortings. Enlargements were represented rights for some genes. *p < 0.05 versus Sca-1^−^ cells and ^°^p < 0.05 versus Sca-1^low+^.

**Figure 3:**
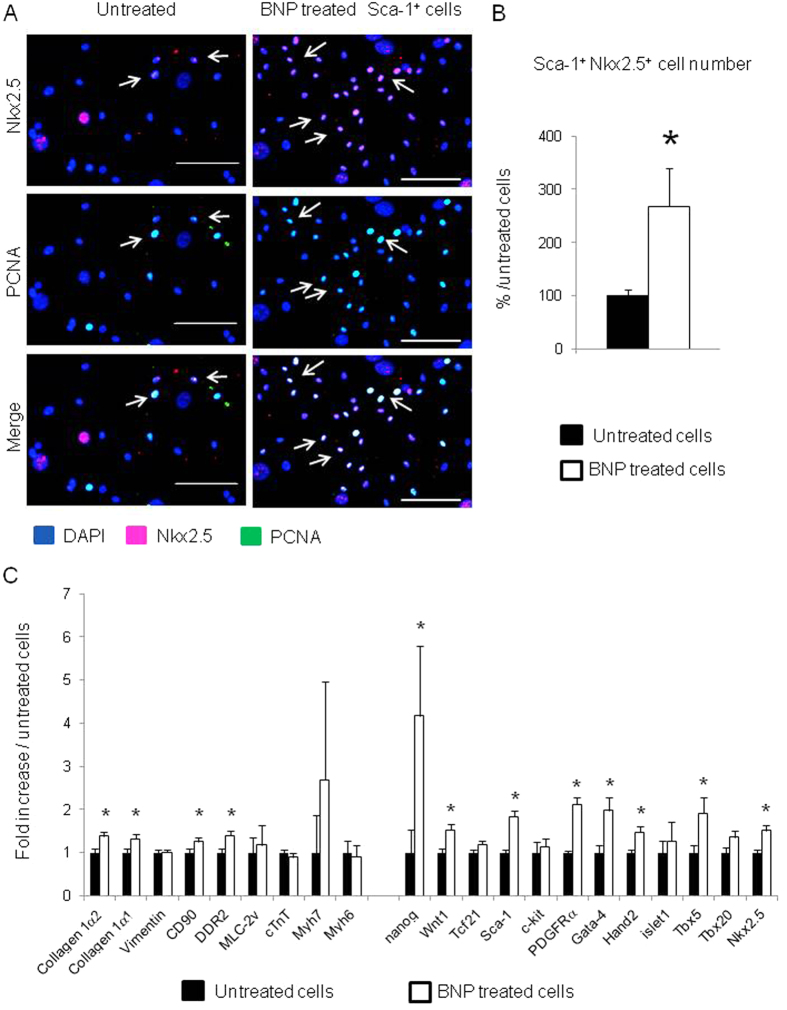
BNP treatment increases the number of Nkx2.5^+^ cells among sorted Sca-1^high+^ cells. (**A**) Representative immunostainings of sorted Sca-1^high+^ cells treated or not with BNP for up to 11 days and stained with antibodies against Nkx2.5 (pink), Proliferating Cell Nuclear Antigen (PCNA) (green) and DAPI (blue). White arrows represent proliferating Nkx2.5^+^ cells. Scale bars represent 100 μm. (**B**) Quantification of the number of Nkx2.5^+^ cells among Sca-1^high+^ cells treated or not with BNP for up to 11 days. The number of Nkx2.5^+^ cells was related to the total number of nuclei stained with DAPI. n = 5–6 different experiments and in total 3607 cells in untreated and 3867 cells in BNP treated cells were evaluated for the Nkx2.5 expression. (**C**) Quantitative polymerase chain reaction in untreated (n = 13 different experiments) and BNP treated Sca-1^high+^ sorted cells (n = 14). Results are expressed as 2^−ΔΔCT^ with untreated cells as reference. (**B** and **C**) Results expressed as fold-increase above the results in untreated cells. All the results are means ± SEM. *p < 0.05.

Among Sca-1^+^ cells, immunostainings highlighted the presence of a subset of cells expressing BNP receptors and the nuclear transcription factor Nkx2.5. Interestingly, in our cell culture conditions, Nkx2.5 mRNA expression was enriched 6 and 2 fold in Sca-1^high+^ cells versus Sca-1^−^ and Sca-1^low+^ cells, respectively (Fig. 2). The effect of BNP on Sca-1^high+^ cells was investigated by stimulating the cells for up to 11 days and by performing immunostainings against Nkx2.5. 27% ± 7% of the BNP treated Sca-1^high+^ cells expressed the protein Nkx2.5 versus 10% ± 1% of the untreated ones (p = 0.02) (Fig. 3A and B). Accordingly, mRNA levels coding for Sca-1 (x1.8) and Nkx2.5 (x1.5) were increased in BNP treated Sca-1^high+^ cells compared to untreated cells. mRNAs coding for genes involved in pluripotency (nanog x4.1) and in cardiogenesis (Gata-4 x2, Hand2 x1.5, Tbx5 x1.9) were also increased after BNP treatment as well as mRNAs coding for PDGFRα (x2.1) and Wnt1 (x1.5) (Fig. 3C).

More PCNA^+^ Nkx2.5^+^ cells were detected in the BNP treated Sca-1^high+^ cells than in untreated ones, demonstrating that BNP was able to stimulate Sca-1^+^ Nkx2.5^+^ cell proliferation (Fig. 3A, white arrows). However, we cannot exclude that BNP could also stimulate the differentiation of Sca-1^+^ Nkx2.5^−^ cells into Sca-1^+^ Nkx2.5^+^ cells. Both mechanisms could lead to increase the number of Sca-1^+^ Nkx2.5^+^ cells *in vitro*.

*In vivo,* in the hearts of neonatal control mice, rare Sca-1^+^ Nkx2.5^+^ cells were localized in small niches, whereas in the hearts of BNP injected mice, the niches expanded and more Nkx2.5^+^ Sca-1^+^ cells appeared (Supplemental Figure 3A). The difference in the number of Sca-1^+^ Nkx2.5^+^ cells was, however, not statistically significant (Supplemental Fig. 3B). In infarcted hearts, higher number of Sca-1^+^ Nkx2.5^+^ cells was detected in the infarcted area of hearts injected with BNP (MI + BNP) compared to hearts injected with saline (MI) (Supplemental Fig. 3C). These results suggested that BNP treatment generated also increased number of Sca-1^+^ Nkx2.5^+^ cells *in vivo*.

### Sca-1^high+^ cells are mainly fibroblasts expressing cardiogenic factors

Sca-1 protein was expressed on several cell types, such as endothelial cells, fibroblast or CPCs. Furthermore, Sca-1^+^ Nkx2.5^+^ cells were identified, according recent data, as being fibroblasts^10^ or CPCs^10^,^14^,^22^. Thus, we tried to identify the Sca-1^high+^ cells whose proliferation was stimulated by BNP treatment (Fig. 1E). Sorted Sca-1^high+^ cells were characterized immediately after sorting for their mRNA expression of genes coding for known fibroblast, cardiac and endothelial or vascular markers. Molecular expressions of pluripotency, mesenchymal stem cell markers, epicardial and cardiac transcription factors were also analyzed (Fig. 2). The gene expression profile was the same between the Sca-1^−^, the Sca-1^low+^ and the Sca-1^high+^ cells with high expression of fibroblast markers, including collagen 1α2, collagen 1α1, vimentin, CD90 (Thymus Cell antigen-1) and discoidin domain receptor 2 (DDR2). Cardiomyocyte or vascular specific markers were not expressed or expressed at a very low level, excepted for Troponin T. mRNA expression of epicardial transcription factor 21 (Tcf21) and Wilms tumor-1 (Wt1) demonstrated the epicardial origin of the cells. The mesenchymal stem cell marker PDGFRα as well as cardiogenic transcription factors (Gata-4, Hand2, Tbx20 and Nkx2.5) were expressed in these three cell subsets. Low or no expression of pluripotency genes (oct4, nanog, Sox2 and Brachyury) and of c-kit was observed.

Sca-1^high+^ cells showed an enrichment in mRNAs coding for Sca-1 (x3.8), PDGFRα (x3.9) and for cardiac transcription factors in particular Tbx5 (x3.9) and Nkx2.5 (x1.8) when compared to unsorted NMCs (Fig. 2). At the protein level, 5% of the Sca-1^high+^ cells expressed c-kit, 8% the NPR-A and 13% the NPR-B receptors. 44% of the Sca-1^high+^ cells were PDGFRα^+^ and we detected rare expression of CD45 (0.6%) and no expression of CD31 (Supplemental Fig. 4).

### A subset of Sca-1^high+^ cells is able to differentiate into cardiomyocytes, endothelial and smooth muscle cells

**Figure 4:**
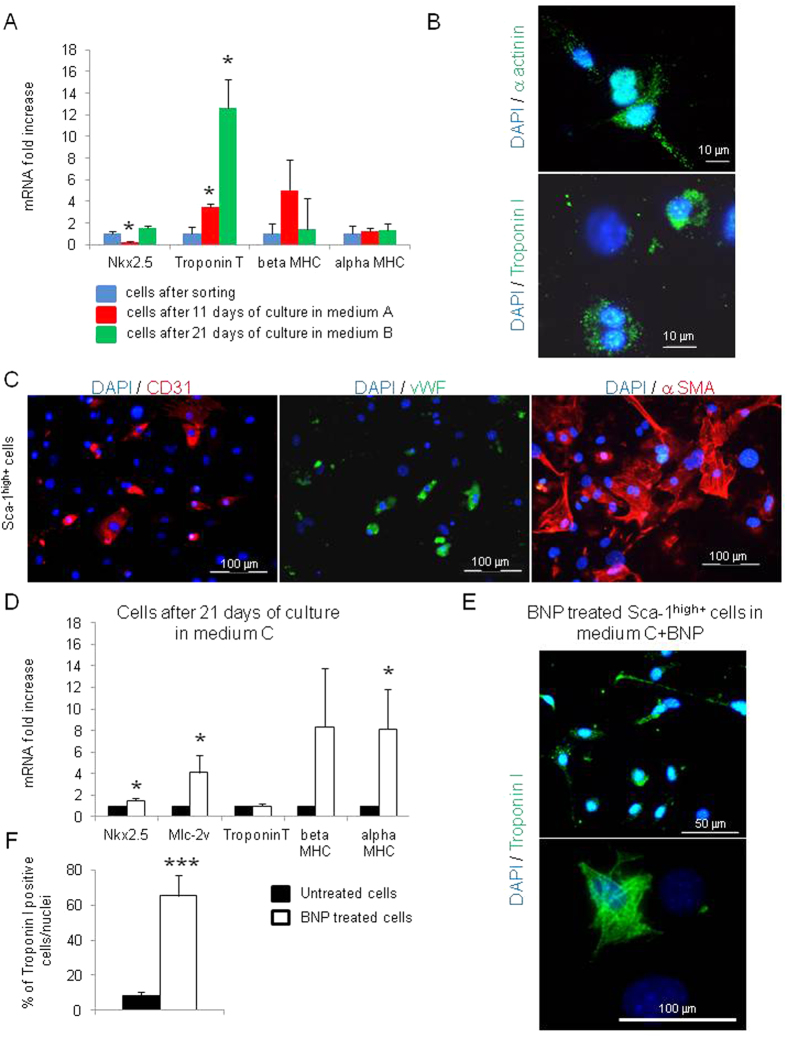
Pluripotency of sorted Sca-1^high+^ cells. (**A**) Quantitative relative expression of mRNAs coding for cardiomyocyte specific genes (Nkx2.5, ventricular Myosin Light Chain, Troponin T, beta and alpha Myosin Heavy Chain) in cells cultured 11 or 21 days in two different media. Results expressed as fold-increase above the levels in cells directly after sorting, n = 4–8 different experiments per group. (**B**) Representative pictures of cells cultured 21 days in medium B and expressing α actinin and Troponin I proteins. (**C**) Representative immunostainings of cells cultured 7 days in medium A and stained for the expression of CD31, von Willbrand Factor (vWF) and smooth muscle actin. (**D**) Quantitative relative expression of mRNAs coding for cardiomyocyte specific genes in cells cultured 21 days in differentiating medium C with or without BNP. Results expressed as fold-increase above the levels in untreated cells. n = at least 7 different experiments. (**E**) Representative immunostainings of cells cultured 21 days in medium C with BNP and stained with Troponin I (green) antibody and DAPI (blue). (**F**) The number of cells positive for Troponin I were related to the number of nuclei. n = 4–7 different experiments and in total, 1313 cells in BNP treated cells and 360 cells in untreated cells were evaluated for the Troponin I expression. (**A**,**D**,**F**) All the results are means ± SEM, *p < 0.05.

Altogether our results demonstrated that the majority of the Sca-1^high+^ cells were fibroblasts expressing cardiogenic transcription factors as previously described by Furtado *et al*.^10^. However, unlike Furtado *et al*., we detected in our Sca-1^high+^ cells directly after sorting, mRNA coding for Troponin T, suggesting the presence of CPCs able to differentiate into cardiomyocytes (Fig. 2A). We thus tested the “differentiation” potential of the Sca-1^high+^ cells *in vitro*. During cell culture in two different media, Sca-1^high+^ cells increased Troponin T mRNA level (Fig. 4A). 8.3% ± 2% of the cells expressed the Troponin I protein which remained located in the nucleus and in the cytoplasm under an unorganized form (Fig. 4B), demonstrating that the cells were at the first step of differentiation^23^. When cultured in EGM-2, Sca-1^high+^ cells were also able to differentiate into endothelial cells, expressing the CD31 protein and the von Willbrand Factor, and into smooth muscle cells (Fig. 4C), demonstrating the multipotency of a subset of Sca-1^high+^ cells. Furthermore, after BNP addition, mRNAs coding for Nkx2.5, Mlc-2v and MHC genes were upregulated and the number of cells expressing the Troponin I was 7.8 fold increased (Fig. 4D and F). In the majority of the cells, Troponin I was located in the nucleus and in the cytoplasm but remained disorganized (Fig. 4E). However, some cells exhibited structurally well organized Troponin I (Fig. 4E), demonstrating that BNP treatment could induce the differentiation of Sca-1^high+^ cells into mature cardiomyocytes a step further. This is consistent with our previous results demonstrating the presence of newly formed cardiomyocytes in the hearts of BNP treated mice^16^. BNP addition didn’t modify the rate of cell differentiation into endothelial or smooth muscle cells. Thus, among Sca-1^high+^ cells, we highlighted the presence of multipotent CPCs able to differentiate into several cell lineages.

### BNP acts via NPR-B to stimulate Sca-1^+^ cell proliferation

**Figure 5:**
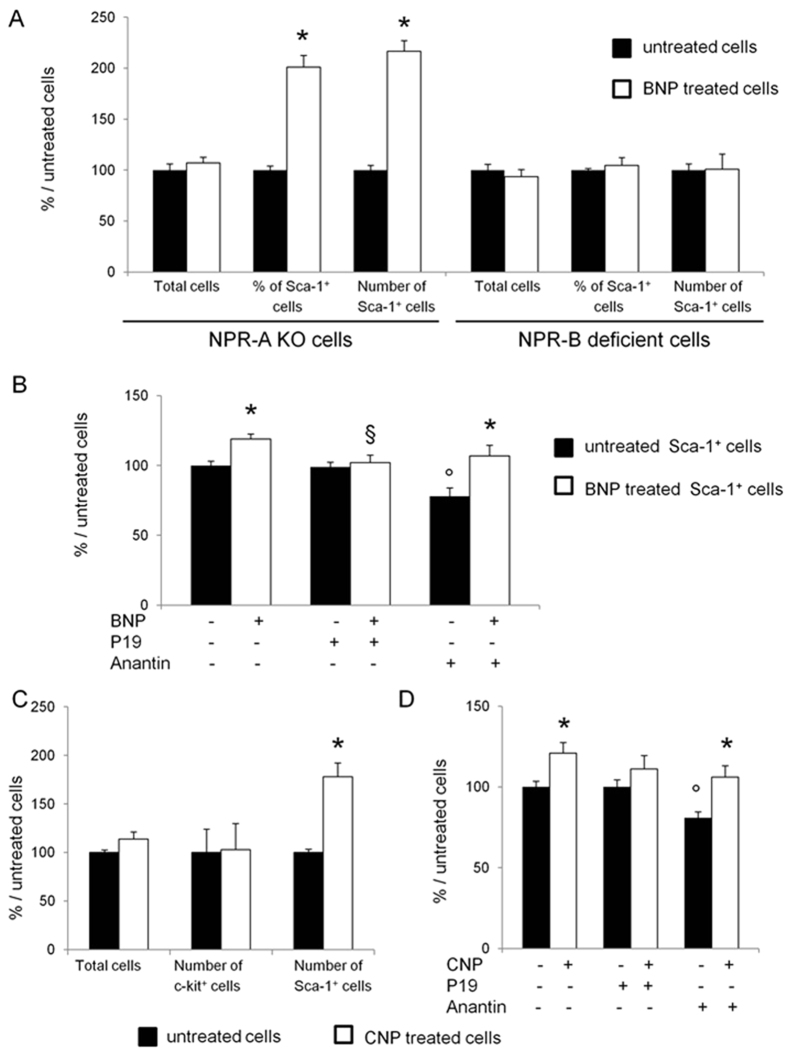
BNP binds to NPR-B to stimulate Sca-1^high+^ cell proliferation. (**A**) Non-myocyte cells (NMCs) isolated from the hearts of neonatal NPR-A or NPR-B deficient mice were cultured with and without BNP. The total number of cells, the percentages of Sca-1^+^ cells obtained by flow cytometry analysis as well as the number of Sca-1^+^ cells were determined 11 days after the onset of BNP treatment. n = 9 different experiments for NPR-A and NPR-B deficient cells. (**B** and **D**) Sorted Sca-1^high+^ cells isolated from wild type hearts were cultured with BNP or CNP in presence or not of NPR-B receptor antagonist (P19, 0.5–1 microM) and of an ANF antagonist (Anantin, 0.2 microM). The number of cells was counted after 9–11 days of culture and the results were related to untreated cells. n = at least 4 different experiments. (**C**) NMCs isolated from wild type hearts were treated with C-natriuretic peptide (CNP) for up to 11 days. The total number of cells as well as the number of c-kit^+^ and Sca-1^+^ cells were determined after counting and flow cytometry analysis. n = 6 different experiments (**D**). (**A**–**D**) Results expressed as fold-increase above the results in the related untreated cells. All the results are means ± SEM. *p < 0.05 versus the related untreated cells, ^°^p < 0.05 versus the untreated cells without inhibitors, ^§^p < 0.05 versus the BNP treated cells without inhibitors.

We then investigated the signaling pathway by which BNP stimulates the Sca-1^high+^ cell proliferation. To determine which is the receptor involved, NMCs isolated from neonatal hearts of mice deficient either for the NPR-A (NPR-A KO mice) or for the NPR-B (NPR-B deficient mice) receptor, were cultured and treated with or without BNP. At time of stimulation with BNP (i.e. 3 days after isolation), NPR-B^+^ Sca-1^+^ or NPR-A^+^ Sca-1^+^ cells were identified in NPR-A KO or NPR-B deficient NMCs in the same proportion than in control cells (B6 + cells) (Supplemental Table S1). After 11 days, BNP stimulation led to increased Sca-1^+^ cell percentage (+101 ± 8%, p < 0.001) and number (+116.5 ± 10.5%, p < 0.001) in NPR-A but not in NPR-B deficient NMCs (Fig. 5A). Accordingly, no increase of the Sca-1 mRNA level was detected in BNP treated NPR-B deficient cells (Supplemental Fig. 5). Sorted Sca-1^+^ cells were also treated with BNP in presence of P19 (a NPR-B antagonist) or Anantin (an ANF antagonist) (Fig. 5B). Blocking the NPR-B receptor with P19 inhibited Sca-1^+^ cell proliferation induced by BNP, whereas blocking the NPR-A receptor with Anantin had no effect (+37% in Sca-1^+^ cells treated with BNP and Anantin compared to Anantin treated cells). Interestingly is the decrease of the number of cells treated with Anantin alone compared to untreated cells (−19%, p < 0.01), suggesting that NPR-A receptor is involved in the cell proliferation in the absence of any stimulation (Fig. 5B). Finally, to assess for the implication of NPR-B receptor, NMCs were treated with the C-related Natriuretic Peptide (CNP, which binds to NPR-B but not to NPR-A) (Fig. 5C). CNP treatment led to increased number of Sca-1^+^ cells (+78 ± 14%, p = 0.002) but not of c-kit^+^ cells (Fig. 5C). Furthermore, CNP stimulation of Sca-1^+^ cell proliferation was abolished by P19 and not by Anantin, demonstrating that CNP was also bound to NPR-B (Fig. 5D). This confirmed preliminary *in vivo* and *in vitro* data demonstrating that CNP injections in neonatal mice increased the number of Sca-1^+^ and Sca-1^+^ Nkx2.5^+^ cells in the heart (Supplementary Fig. 6).

### BNP treatment activates the protein kinase G and the p38 MAP kinase

**Figure 6:**
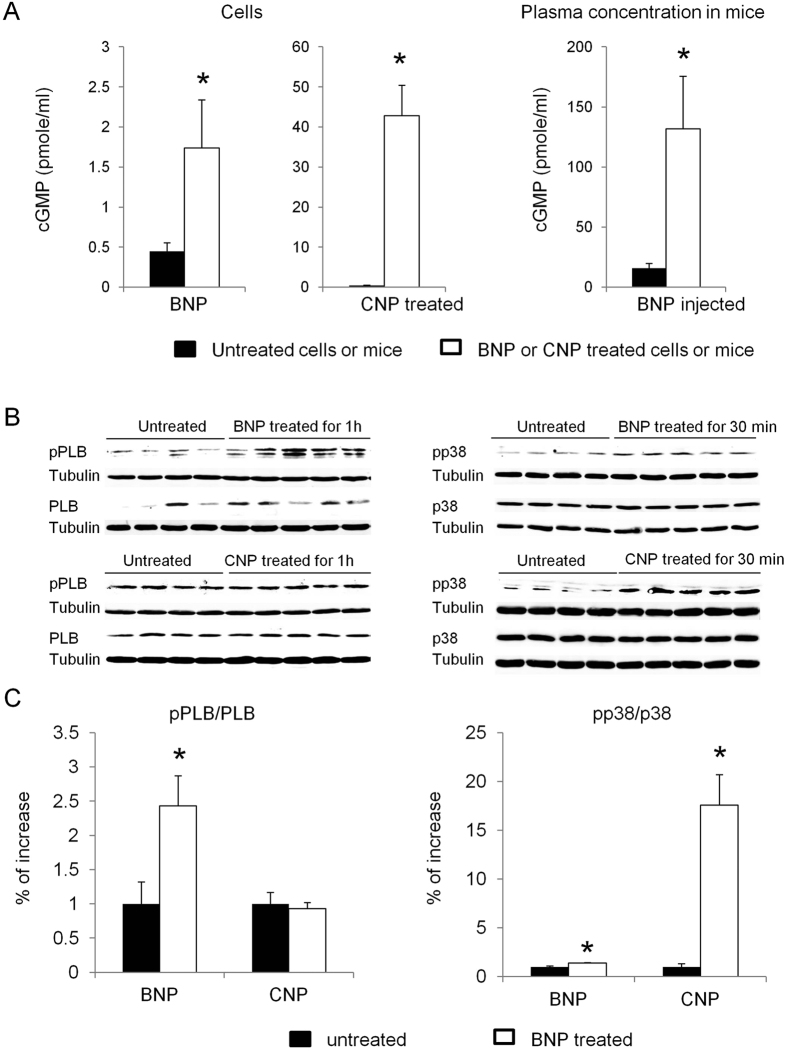
BNP treatment increases cGMP level and activates the protein Kinase G and the p38 MAP kinase. (**A**) cGMP levels were measured in the non myocyte cell supernatants (n = 4–7) and in the EDTA treated plasma of mice (n = 4 mice per group) 1h after BNP or CNP treatment (1 μM). (**B**) Representative western blots of sorted Sca-1^high+^ cells isolated from B6^+^ neonatal hearts and stimulated with or without BNP or CNP (1 μM both). Blots were stained with antibodies against phospho phospholamban (pPLB), phospholamban (PBL), phospho p38 (pp38), p38 and Tubulin (used as loading control). Only the bands at the adequate molecular weight were represented here: tubulin 55 kDa, pp38 and p38 about 43 kDa, pPLB between 21 and 26 kDa and PLB 25 kDa. (**C**) Quantification of the data from western blot analysis expressed relatively to the average of untreated cells. Sorted Sca-1^high+^ cells were or not treated 1h with BNP or CNP. n = 4–6 different experiments. All the results are means ± SEM, *p < 0.05 versus untreated cells.

The signaling pathway by which BNP acts on Sca-1^+^ cell proliferation was investigated. First we evaluated whether BNP treatment *in vitro* but also *in vivo* increased cGMP levels. One hour after BNP treatment or injection, cGMP level was increased 4 and 8 fold in the treated cells or mice, respectively (Fig. 6A). Secondly, western blot analyses were performed on sorted Sca-1^high+^ cells, treated or not with BNP to assess for PKG activation. The ratio phospho phospholamban (pPLB)/phospholamban (PLB) was used to evaluate PKG activation on BNP treated cells (Fig. 6B). BNP treatment of Sca-1^high+^ cells induced rapidly an increase of this ratio (x2.4) after 1 h, (p = 0.01), and also of the pp38/p38 ratio (x1.4) after 30 min, (p = 0.0006) (Fig. 6B,C). To determine whether p38 MAP kinase phosphorylation was linked to PKG activation, a PKG inhibitor was added to BNP treated NMC culture and reduced PLB and p38 phosphorylations after BNP treatment by 38 and 50%, respectively (Supplemental Fig. 7). Addition of a p38 MAP kinase inhibitor in BNP treated cells reduced p38 phosphorylation by 31% but didn’t affect PLB phosphorylation (Supplemental Fig. 7), demonstrating that at least, a part of p38 phosphorylation was dependent on PKG activation.

**Figure 7:**
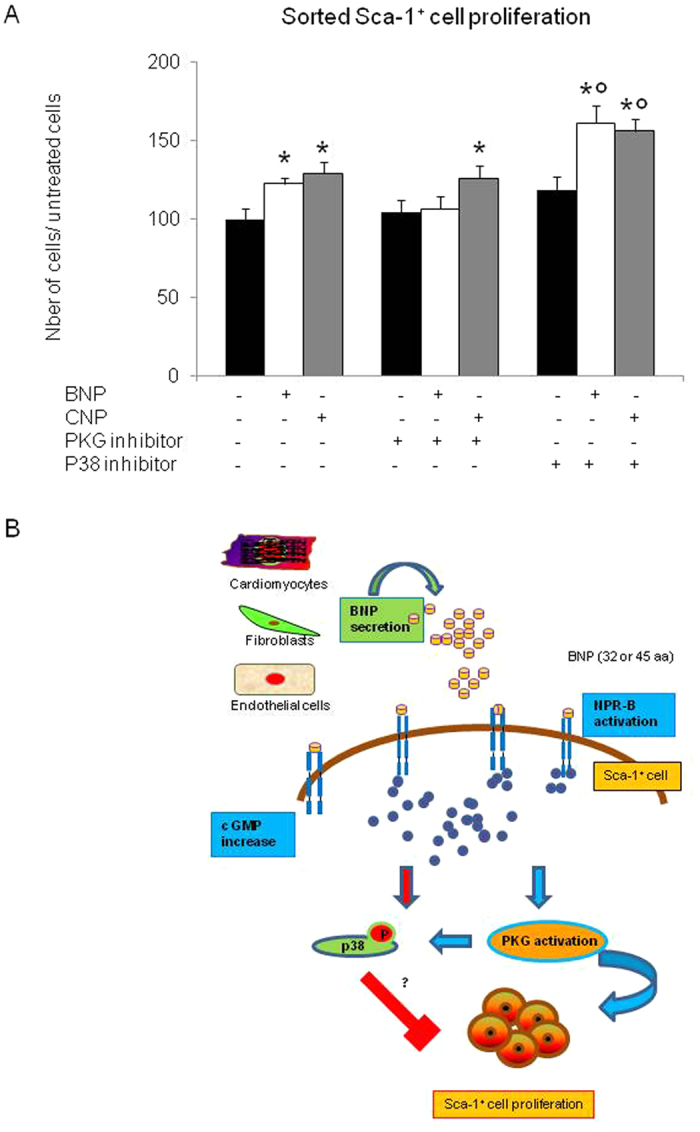
Schematic representation of BNP signaling pathway leading to Sca-1^+^ cell proliferation. (**A**) Number of sorted Sca-1^high+^ cells after 11 days of culture with or without BNP or CNP and PKG or p38 inhibitor. n = at least 6 different experiments per group. The data were related to the average of untreated cells. All the results are means ± SEM, *p < 0.05 versus untreated cells and ^°^p < 0.05 versus BNP or CNP treated cells. (**B**) Summary of the hypothetical signaling pathway induced by BNP to stimulate Sca-1^+^ cell proliferation. BNP secreted by cardiac cells binds to NPR-B on Sca-1^+^ cells which increases the cGMP level and activates the protein kinase G (PKG). Furthermore, BNP phosphorylates the p38 MAP kinase. Addition of the SB203580 increases cell proliferation.

At the cellular level, adding a PKG inhibitor to BNP treated sorted Sca-1^high+^ cells inhibited the BNP effect on their cell proliferation, whereas adding SB203580 to these BNP stimulated cell cultures increased the number of Sca-1^high+^ cells (+34% versus BNP treated cells, p = 0.004) (Fig. 7A).

### The other members of the natriuretic peptide family, ANP and CNP, modulate also the Sca-1^+^ cell proliferation

The CNP, as the BNP, induced Sca-1^+^ cell proliferation via NPR-B. Both peptides increased the level of cGMP in the cells (Figs 5D and 6A). Furthermore, adding SB203580 to the CNP-treated cell cultures increased the number of Sca-1^high+^ cells (+21% compared to CNP treated cells, p = 0.015) (Fig. 7A). However, in contrast to BNP treatment, adding a PKG inhibitor to CNP-treated Sca-1^+^ cells had no effect on their proliferation, suggesting that CNP acted via a PKG-independent signaling pathway (Fig. 7A).

The effect of the atrial natriuretic peptide (ANP) on the Sca-1^+^ cell proliferation is less evident than this of BNP or CNP (Supplementary Fig. 8). Although the number of Sca-1^+^ cells was increased (+22%) after 11 days of ANP treatment, this was statistically not significant (p = 0.09). Both receptors, NPR-A and NPR-B, seemed to be involved and were able to activate PKG via the increase of cGMP. The p38 MAP kinase was not activated by ANP (Supplementary Fig. 8B and C).

## Discussion

The main finding of this study is the identification of a signaling pathway able to modulate the proliferation of the “endogenous” Sca-1^+^ cells in the heart. Thus, we demonstrated that (1) BNP treatment increased the number of Sca-1^+^ cells by stimulating their proliferation and their differentiation into Sca-1^+^ Nkx2.5^+^ cells. (2) BNP induced Sca-1 protein expression on Sca-1^−^ cells. (3) The isolated Sca-1^+^ cells were characterized as a heterogenous cell population composed of fibroblasts and multipotent stem cells. (4) BNP stimulation of Sca-1^+^ cell proliferation was mediated by NPR-B binding, increased cGMP level and PKG activation. (5) CNP was also able to stimulate Sca-1^+^ cell proliferation but via a PKG-independent signaling pathway.

The first important finding of our work is the identification, among NMCs, of the cells responding to BNP. In previous work, we demonstrated that BNP treatment stimulated the proliferation of Nkx2.5^+^ α actinin^−^ NMCs and the formation of new cardiomyocytes^16^. We thus hypothesized that BNP could modulate CPC’s fate^16^. That is why we focused our attention on BNP effect on the c-kit^+^ and the Sca-1^+^ cells isolated from neonatal murine hearts. Both cell subsets expressed BNP receptors and were able to respond to BNP stimulation (Supplemental Table S1).

In our culture conditions, BNP treatment increased the number of Sca-1^+^ cells by two mechanisms: the stimulation of the Sca-1^+^ cell proliferation and the induction of the Sca-1 protein expression on Sca-1^−^ cells (Fig. 1). Furthermore, among the Sca-1^+^ cells, BNP treatment increased the number of cells expressing the nuclear transcription factor Nkx2.5 (Fig. 3). Again, it seemed that BNP was able to stimulate the proliferation of the Sca-1^+^ Nkx2.5^+^ cells (Fig. 3A). However, an effect of BNP on cell differentiation (i.e. Nkx2.5 expression by Nkx2.5^−^ cells) cannot be excluded. In fact, accordingly to our previous work, we showed again in Fig. 4D–F that BNP treatment was able to push the differentiation of CPCs into the cardiogenic lineage (with increased mRNA levels coding for Nkx2.5, Mlc-2v and alpha MHC). Altogether, these results suggested that BNP could act on cardiac cell proliferation and on cell differentiation, as previously published^16^.

Whatever the mechanisms, the fact that BNP treatment is able to increase the number of Sca-1^+^ cells and especially the number of Sca-1^+^ Nkx2.5^+^ cells is of high interest for cardiac cellular therapies aimed to induce heart regeneration. Sca-1^+^ Nkx2.5^+^ cells were for a long time considered as cardiac precursor cells, able to differentiate into mature and functional cardiomyocytes^2^,^6^,^22^. However, recently, Furtado *et al*. demonstrated that around 70% of the cardiac fibroblasts expressed Sca-1 and half of the Sca-1^+^ fibroblasts are able to express Nkx2.5^10^, demonstrating that even the Sca-1^+^ Nkx2.5^+^ cells are a mixed cell population. This is clearly the case in our work. We isolated Sca-1^+^ cells which expressed “fibroblast” markers and cardiogenic precursor markers, suggesting that our Sca-1^+^ cells were similar to the fibroblasts described by Furtado *et al*., excepted that we found, in our cells, expression of Troponin T (at the mRNA level) and Troponin I (at the protein level) (Figs 2 and 4). Troponin expression in our Sca-1^+^ cells highlighted the presence of multipotent cells able to differentiate into several cell types (Fig. 4). Indeed, until now, cardiac fibroblasts have not been shown to be able to differentiate spontaneously into cardiomyocytes *in vitro* or *in vivo*^10^. However, these cells have the capacity to “de-differentiate “*in vitro* (by genetic manipulations)^24^,^25^, thus we cannot exclude that this process doesn’t occur spontaneously in some conditions *in vivo*.

Even if our Sca-1^+^ cells were not “pure” precursor cells, BNP effect on these cells or on a part of these cells must contribute to its “protective” effect *in vivo*^16^,^26^,^27^,^28^. In this work, we showed in Supplemental Fig. 3 that BNP injections into neonatal or adult mice after myocardial infarction, led to increased number of Sca-1^+^ Nkx2.5^+^ cells. This result is in agreement with the report of Hsueh *et al*. demonstrating that the Sca-1^+^ cells differentiated into cardiomyocytes after MI^2^. If we assume that a part of these Sca-1^+^ cells are CPCs, able to differentiate into cardiomyocytes after BNP treatment as already demonstrated^16^, what is the role of the Sca-1^+^ fibroblasts in these hearts? Increased fibrosis would be detrimental for the BNP treated hearts and we didn’t observe in BNP treated hearts, increased fibrosis (personal communication). Thus, the role and function of the fibroblasts, expressing Sca-1 together with cardiogenic markers, have to be characterized. Interestingly, specific deletion of Tbx20 in these fibroblasts affected cardiomyocyte differentiation during heart development^10^. As suggested by others, this specific subset of fibroblasts could support heart regeneration by secreting some specific factors such as the Fibroblast growth factor 2 or Prostaglandin E2, which could promote the differentiation of endogenous Sca-1^+^ cells into cardiomyocytes after MI^2^,^22^.

In this work we tried also to identify a signaling pathway able to induce Sca-1^+^ cell proliferation. Surprisingly, we noticed that the 3 natriuretic peptides (BNP, CNP and ANP) were able to induce increased number of Sca-1^+^ cells after 11 days of treatment. However the mechanisms used by these peptides on Sca-1^+^ cells differed. BNP and CNP effects were clearly inhibited by NPR-B antagonist and not by Anantin, demonstrating that BNP and CNP stimulated Sca-1^+^ cell proliferation only via NPR-B binding (Fig. 5B and D). In contrast, ANP stimulation on Sca-1^+^ cell proliferation was inhibited by both receptor antagonists, suggesting that ANP could act via NPR-B but also via NPR-A (Supplementary Fig. 8B). Furthermore, NPR-A seemed to be involved in the proliferation of the unstimulated Sca-1^+^ cells as Anantin inhibited this proliferation by 20% (Fig. 5B and D). Further experiments were required to determine more precisely the role of NPR-A in ANP-stimulated Sca-1^+^ cells.

NPR-B was shown here to be a key element in the stimulation of the Sca-1^+^ cell proliferation by the natriuretic peptides. The involvement of this receptor was particularly interesting as NPR-B has been shown to be the major natriuretic peptide receptor in the failing mouse heart after transaortic constriction^29^.

BNP and CNP bound to NPR-B, increased cGMP levels and activated or not the protein kinase G (PKG) to stimulate the proliferation of the Sca-1^+^ cells (see Fig. 7B). Indeed our results suggested that CNP stimulated Sca-1^+^ cell proliferation via a PKG-independent mechanism (Figs 6 and 7). We didn’t identify yet the mechanism by which CNP stimulated the Sca-1^+^ cell proliferation. However, increased cGMP level was shown to modulate the activity of several proteins involved in cellular mechanisms and in cardioprotection, such as the Protein kinase A or the phosphodiesterases 2 and 3^30^.

In parallel, BNP and CNP’s binding on the Sca-1^+^ cells activated the p38 MAP kinase. Interestingly, the inhibition of this protein by high doses of SB203580 increased the number of Sca-1^+^ cells after BNP and CNP treatment. Thus, the “inhibitory” role of the p38 MAP kinase signaling pathway on Sca-1^+^ cell proliferation has also to be studied in more details.

The identification of CNP as a stimulatory factor of Sca-1^+^ cell proliferation is also of great interest. Indeed, CNP is secreted inside the heart at very low levels when compared to BNP or ANP levels^31^. However, its concentration increases after shear stress, hypoxia, exercise preconditioning and/or stimulation with several cytokines such as the Transforming growth factor β^32^,^33^,^34^. CNP induces vasodilation and exerts anti-proliferative effects on fibroblasts and on vascular smooth muscle cells^35^,^36^. Furthermore, CNP treatment reduces fibroblast collagen production and stimulates osteoblast and endothelial cell differentiation^37^,^38^. *In vivo*, CNP has been shown to be cardioprotective by improving left ventricle capacities after heart failure in dogs, by preventing cardiomyocyte hypertrophy in infarcted mouse hearts and by increasing cardiomyocyte relaxation and inotropic response in failing rat hearts^34^,^39^,^40^,^41^. Here in this report we showed that CNP is able to stimulate the proliferation of the cardiac Sca-1^+^ cells. Whether CNP is also able to induce their differentiation into several cell types is under evaluation. Preliminary *in vitro* experiments demonstrated that CNP treated non-myocyte cells were able to increase mRNA levels of cardiomyocyte specific genes (Nkx2.5, Gata-4, Mlc-2v, Myh6) (Supplementary Fig. 6C). Furthermore, CNP treatment *in vivo* led to increased number of Sca-1^+^ cells and even of Sca-1^+^ Nkx2.5^+^ cells in hearts of young CNP injected mice. Further experiments are in progress to confirm these results and to evaluate CNP effect on Sca-1^+^ cells after myocardial infarction in adult mice.

Despite some limitations of this study (we used high doses of natriuretic peptides, we didn’t measure plasma BNP levels and we detected a huge increase of cGMP *in vivo*), the identification of the signaling pathways by which BNP modulates the fate of the endogenous Sca-1^+^ cells is of high importance for optimizing further clinical use of the natriuretic peptides in patients suffering from heart diseases. It is important here to mention that the human Sca-1 homologue doesn’t exist. However, in human fetal hearts, the use of an anti-mouse Sca-1 antibody allowed to characterize and isolate “Sca-1^+^” cells with all the features of stem cells. In particular, these “sorted human Sca-1^+^ cells” were able to differentiate into cardiomyocytes^42^,^43^. In this report we identified a signaling pathway able to increase the number of the multipotent Sca-1^+^ cells in the heart. Whether these cells are able to contribute to heart regeneration remains to be proved.

## Material and Methods

### Mice

All animal procedures were performed conform the guidelines from Directive 2010/63/EU of the European Parliament on the protection of animals used for research and are also in accordance with the recommandations of the U.S. National Institutes of Health Guide for the Care and Use of Laboratory Animals (National Institutes of Health publication 86-23, 2011). All our experiments were approved by the Swiss animal welfare authorities (autorisation VD2765 and VD2321). C57BL/6 mice (Wild Type mice, WT) were purchased from Janvier (Le Genest-Saint-Isle, France) and were housed in the animal facility. The NPR-A (−/−) mice were kindly provided by Dr Feng Li and Prof Nobuyo Maeda (Chapel Hill, North Carolina, US)^44^. The NPR-B deficient mouse strain (C57BL/6J-*Npr*2^*slw*^) was generated as heterozygous mice in the Laboratory of Animal Resource Bank at National Institute of Biomedical Innovation, Osaka, Japan^45^. Both colonies (NPR-A KO and NPR-B deficient mice) were established in our animal facility. Neonatal mice were sacrificed 1–2 days after birth and adult mice were sacrificed after 12 weeks.

### Cell isolation and proliferation

NMCs were isolated as previously described^22^. All cells were used after one passage to avoid the changes in the cell phenotype and/or genetic profile. Thus, after isolation, at confluence, the cells were diluted 1:3 before to be either sorted for their Sca-1 expression or directly treated with BNP (1 microM murine BNP (recombinant mouse C 1–45 peptide; American Peptide Co)), CNP (1 microM C-type Natriuretic Peptide (recombinant CNP-22 peptide; Phoenix Pharmaceuticals, Inc)) or ANP (1 microM, Phoenix Pharmaceuticals, Inc). Cells were cultured for up to 11 days in 10% horse serum and 5% foetal bovine serum medium. We chose to count and analyze the cell phenotype at two stages: 4 days because we demonstrated previously that BNP effect on NMCs is detectable at this time and 11 days because the cells reached confluence^16^. A dose response curve for BNP and CNP effect on cardiac cells was also previously established^16^. Sorted Sca-1^high+^ cells were also directly treated with BNP or CNP for up to 11 days after cell sorting. To determine which receptor is involved in BNP effect on sorted Sca-1^+^ cells, cultures were performed in presence or not of the NPR-B receptor antagonist P19 (Phoenix Pharmaceuticals, 0.5 microM) or the ANF antagonist Anantin (Bachem, 0.2 microM) which were added 30 min before BNP, CNP or ANP treatment. To determine by which signaling pathway BNP acts on Sca-1^high+^ cells to induce their proliferation, a PKG inhibitor (Guanosine 3′, 5′-cyclic Monophosphorothioate, β-Phenyl-1, N2-etheno-8-bromo-Rp-Isomer, Sodium Salt (Calbiochem) 0.1 microM)) or a p38 MAP kinase inhibitor (SB203580 (Enzo) 10 microM) was added to the cell culture 15 min or 1 hour, respectively, before the BNP treatment. Media were changed twice a week.

### Flow cytometry analysis and Cell sorting

Cultured unsorted NMCs as well as neonatal or adult NMCs (directly after isolation) were stained with several antibodies listed in Supplemental Table S2. Stainings with isotype control (the same dilution as the referred antibodies) were performed for all stainings. Single, double or triple stainings were performed and analyzed using a Gallios Flow Cytometer (Beckman Coulter). For cell sorting, NMCs were stained with the rat anti-Sca-1^−^PeCy5 antibody. The cells were sorted with the MoFlo Astrios Flow Cytometer System (Beckman Coulter) depending on their Sca-1 expression. After flow cytometry sorting, selected cells were cultured as unsorted NMCs in the proliferation medium with or without BNP as described above.

### Immunohistology

Neonatal and adult hearts were embedded in OCT. Immunostainings were performed on 5 μm heart sections or on sorted or unsorted cells cultured for up to 11 days on coverslips. Tissue sections or cells were fixed 10 min in 2% PFA. The first antibodies were all incubated overnight at 4 °C (Supplemental Table S2). Secondary antibodies were incubated 1 h at room temperature. Nuclei were stained with DAPI. All slides were mounted with Dabco mounting medium (Sigma) and examined with a Nikon eclipse 90i microscope.

### Quantitative RT-PCR

Total RNA was isolated using Trizol (Invitrogen Corp, San Diego, CA, USA). The first strand cDNA was synthetised using the M-MLV reverse transcriptase (Promega Corporate, Wisconsin, USA). Quantitative real time polymerase chain reaction was performed in duplicates using the SYBR Premix Ex Taq polymerase (Takara Bio Inc.) on a ViiA^TM^ 7 Instrument (Applied Biosystems). Results were obtained after 40 cycles of a thermal step protocol consisting of 95 °C (1 s), 60 °C (20 s). The sequences of primers were reported in Supplemental Table S3. Gene expression data were normalized to the expression levels of 18S (ΔCT values). Means of ΔCT or of ΔΔCT values (versus untreated cells) were calculated and results were represented as 2^−ΔCT^ or 2^−ΔΔCT^. Statistics were performed on ΔCT or on ΔΔCT individual values.

### Cell differentiation

Three differentiation media were tested to assess for Sca-1^high+^ multipotency. **Medium A** is composed of EGM-2 medium (Lonza Clonetics, Inc)^46^, **medium B** is composed of MEM Alpha (Invitrogen Corp.), 10% FBS (Invitrogen Corp.), 100 U/ml penicillin G, 100 microg/ml streptomycin (Invitrogen Corp.), 1 μM dexamethasone (Sigma-Aldrich), 50 μg/ml ascorbic acid (Sigma-Aldrich), and 10 mM β-glycerophosphate (Sigma-Aldrich)^22^. To determine whether BNP was able to increase cell differentiation into cardiomyocytes, Sca-1^high+^ cells were cultured in **medium C** (MEM Alpha (Invitrogen Corp.), 10% FBS (Invitrogen Corp.), 100 U/ml penicillin G, 100 microg/ml streptomycin (Invitrogen Corp.)) with or without BNP (5 μg/ml). Media were changed twice a week.

### Western blot

Total proteins were extracted from sorted Sca-1^+^ cells and stained with primary antibodies overnight at 4 °C (Supplemental Table S1). Secondary antibodies were added 2 hours at room temperature. The immunoblot signals were detected and quantified using the Odyssey infrared imaging system (LI-COR Biosciences, Bad Homburg, Germany). All results were corrected for their expression of tubulin.

### cGMP detection level

cGMP level was detected using the cGMP Enzyme Immuno Assay kit Direct (Sigma). Blood or cell supernatants in serum free cultured medium were collected 1 h after BNP injection or treatment. EDTA-plasma and cell supernatants were then processed as recommended in the kit.

### Statistical analysis

All results were presented as mean ± SEM. Statistical analyses were performed using the unpaired Student-T test or Wilcoxon-Mann-Whitney test. The alpha level of all tests was 0.05.

## Additional Information

**How to cite this article**: Rignault-Clerc, S. *et al*. Natriuretic Peptide Receptor B modulates the proliferation of the cardiac cells expressing the Stem Cell Antigen-1. *Sci. Rep.* **7**, 41936; doi: 10.1038/srep41936 (2017).

**Publisher's note:** Springer Nature remains neutral with regard to jurisdictional claims in published maps and institutional affiliations.

## Supplementary Material

Supplementary Data
