# Supplementary material for: Natriuretic Peptide Receptor B modulates the proliferation of the cardiac cells expressing the Stem Cell Antigen-1
Source: Sci Rep. 2017 Feb 9;7:41936. doi: 10.1038/srep41936 (PMC5299447; doi:10.1038/srep41936)
Supplement: Supplementary Data [file srep41936-s1.pdf]

Natriuretic Peptide Receptor B modulates the proliferation of the cardiac cells expressing the Stem Cell Antigen-1.

Stéphanie Rignault-Clerc,[1] Christelle Bielmann,[1] Lucas Liaudet,[2] Bernard Waeber [1] François Feihl ,[1] Nathalie Rosenblatt-Velin [1*]

[1] Unité de Physiopathologie Clinique and [2] Service de Médecine Intensive Adulte, Centre Hospitalier Universitaire Vaudois and University of Lausanne, Switzerland.

* Corresponding author

SUPPLEMENTAL METHODS

Cell isolation and proliferation

Neonatal mice were sacrificed by decapitation with surgical scissors and the hearts removed. Hearts (1-2 days-old) were enzymatically digested with 0.45 mg/ml collagenase II (Worthington, Biochemical Corporation, USA) and 1 mg/ml pancreatin (Sigma-Aldrich). Cardiomyocytes were separated from the non-myocyte cells (NMCs) by two differential plating. NMCs were first cultured **in the proliferation medium**: a 3:1 mixture of DMEM and Medium 199 (Invitrogen Corp, San Diego, CA, USA) supplemented with 10% horse serum (Oxoid), 5% fetal bovine serum (FBS) (Invitrogen), 10 mM Hepes, 100 U/ml penicillin G, and 100 microg/ml streptomycin.

Western blot

Total proteins were extracted from sorted Sca-1$^+$ cells after lysis in a buffer containing Nonidet P-40 0.5%, NaCl 150 mM, Na-orthovanadate 1 mM, NaF 10 mM, Tris-HCL pH=7.5 10 mM, PMSF 1 mM, EDTA pH=8 1 mM, aprotinin 10 μg/ml, leupeptin 10 μg/ml, pepstatin 1 μg/ml. 30 μg of these total proteins were separated by SDS-PAGE, transferred to nitrocellulose membranes (Biorad), and blocked for 1h at room temperature in Odyssey blocking buffer (1/2 in TBS Li-COR Biosciences).

Neonatal and infarcted mice treated with BNP

BNP (1 microg/mouse), CNP (1 microg/mouse), or NaCl was injected intraperitoneally (ip) into 3-4 days old C57BL/6 mice (American Peptide Co, Sunnyvale, CA, US) every 2 days during 2 weeks as previously described [1]. Mice were sacrificed 3 days after the

last injection using ip lethal injection of pentobarbital (150 mg/kg). The breathing arrest was controlled and cervical dislocation was performed.

In adult mice, the left anterior descending coronary artery (LAD) was occluded in 10 weeks-old C57 BL/6 mice to induce MI. Mice were anesthetized by ip injection of ketamine/xylazine/acepromazine (65 mg/kg; 15 mg/kg; 2 mg/kg). Mice were moved to a heated plateform. During surgery, the depth of mouse anesthesia was tested by gentle pinch the legs. Directly after the surgery, NaCl or BNP (1 microg/20g in 20 microl) was injected into the myocardium and thereafter BNP (2 microg/mouse) was injected ip every 2 days. After surgery, temgesic (0.1 mg/kg) was injected subcutaneously to reduce the pain, and injections were performed twice per day for up to 4 days. Mice were sacrificed 10 days after MI induction by lethal injection of pentobarbital (150 mg/kg intraperitoneally). The breathing arrest was controlled and cervical dislocation was performed.

REFERENCE

1. Bielmann, C. *et al.* Brain natriuretic peptide is able to stimulate cardiac progenitor cell proliferation and differentiation in murine hearts after birth. *Basic Res Cardiol* **110**, 455-72 (2015).

Supplemental Table S1. Flow cytometric characterization of Non-Myocyte Cells (NMCs) isolated directly from murine hearts or after cell culture.

Age of mice	strain	n	days after isolation	NPR-A+	NPR-B+	c-kit+	Sca-1+	NPR-A+ /c-kit+	NPR-A+ /Sca-1+	NPR-B+ /c-kit+	NPR-B+ /Sca-1+
Adult	WT	6	0	3.3±1.0	6.3±1.1	6.2±1.1	50.7±2.7	2.1±0.7	1.8±0.2	3.5±0.7	3.7±0.8
Neonatal	WT	6	0	2.9±0.3	5.6±1.1	2.9±0.3	15.3±1.6	2.9±0.3	2.0±0.6	2.7±0.4	4.1±1.4
Neonatal	WT	5	3°	3.8±0.5*	2.6±1.0*	2.3±0.9	49.0±4.2*	0.6±0.2*	3.3±0.4	0.6±0.2*	2.1±0.9
Neonatal	NPR-A $^{-/-}$	3	3	--	1.9±0.2	0.7±0.2	64.2±9	--	--	0.4±0.1	1.9±0.5
Neonatal	NPR-B $^{-/-}$	6	3	2.9±0.7	--	1.3±0.5	52.2±5.6	0.9±0.2	2.3±0.5	--	--

Data represent the percentage of cells positive for the different markers and are expressed as the mean ± SEM of the indicated number of experiments (n). Neonatal and adult non-myocyte cells (NMCs) were analysed directly after isolation (0 days after isolation) or ° 3 days after the first passage at confluence, at the onset of BNP treatment. Cells expressing NPR-A and NPR-B (C57BL/6 or WT cells) were compared to cells expressing either only NPR-B (NPR-A KO mice) or only NPR-A (NPR-B deficient mice). * $p<0.05$ between WT cells after isolation and after 3 days after the first passage.

Supplemental Table S2. Antibodies used in flow cytometry analysis, cell sorting, immunohistology and Western blot analysis.

Flow Cytometry analysis and Cell sorting

rat-anti Sca-1 PE-Cy5 or FITC	1/50	eBiosciences
rat anti-c-kit PE-Cy5 or FITC	1/50	eBiosciences
rat anti-CD31 APC	1/100	eBiosciences
rat anti-PDGFR-α PE	1/80	eBiosciences
rabbit anti-NPR-A	1/50	Abcam
+ donkey anti-rabbit FITC	1/1000	Molecular Probes
goat anti-NPR-B	1/20	Santa Cruz Biotechnology
+ chicken anti-goat FITC	1/500	Molecular Probes

Immunohistology

Primary antibodies			Secondary antibodies		
rabbit anti-NPR-A	1/50	Abcam	horse anti-goat biotin	1/200	Vector
goat anti-NPR-B	1/50	Santa Cruz Biotechnology	horse anti-rabbit biotin	1/200	Vector
goat anti-Nkx2.5	1/20	Santa Cruz Biotechnology	donkey anti-rabbit Alexa 594	1/500	
rabbit anti-Nkx2.5	1/100	Abcam	donkey anti-goat Alexa 594	1/1000	
goat anti-Troponin I	1/100	Santa Cruz Biotechnology	donkey anti-rat Alexa 488	1/1000	
rat anti-Sca-1	1/1000	Abcam	goat anti-rabbit Alexa 594	1/2000	Molecular
rabbit anti-PCNA	1/100	Abcam	donkey anti-rabbit Alexa 488	1/1000	Probes
rat anti-CD31 biotin	1/200	Becton Dickinson	Steptavidine Alexa 594	1/1000	
rabbit anti-von Willbrand Factor	1/500	Dako	Streptavidine Alexa 647	1/1000	
			chicken anti-goat Alexa 488	1/1000	
Rabbit anti-smooth muscle actin	1/200	Abcam			

Western blot analysis

Primary antibodies			Secondary antibodies		
rabbit anti-phospho-phospholamban	1/1000	Millipore	goat anti-rabbit Alexa 680	1/5000	Molecular Probes
mouse anti-phospholamban	1/500	Millipore	Anti-mouse IRDye 800	1/5000 or 1/20000 (for tubulin)	Rockland Immunochemicals
rabbit anti- p38	1/1000	Cell Signaling			
rabbit anti-phospho p38	1/500	Cell Signaling			
Mouse anti-tubulin	1/20 000	Sigma			

Supplemental Table S3: Sequences of primers used in quantitative RT-PCR.

Gene	Sense	Anti-sense	Product size (bp)
**Brachyury**	CCTCCCTTGTTGCCTTAGAGTAGTT	GCAGATTGTCTTTGGCTACTTTGTC	185
**CD90**	TGGGTGCAGCAACTGGAGGC	CTCGGGACACCTGCAAGACTGA	181
**CD31**	GCCTCACCAAGAGAACGGAAGGC	CTGCTTTCGGTGGGGACAGGC	158
**c-kit**	ATCTGCTCTGCGTCCTGTTG	CTGATTGTGCTGGATGGATG	108
**Collagen 1α1**	AATGGCACGGCTGTGTGCGA	AACGGGTCCCCTTGGGCCTT	183
**Collagen 1α2**	GGCCCCCTGGTATGACTGGCT	CGCCACGGGGACCACGAATC	129
**DDR2**	TTCCCTGCCCAGCGAGTCCA	ACCACTGCACCCTGACTCCTCC	181
**eNOS**	GGCTGTGGTAGTTAGGGCATC	AGGTTTGGGTTGGGCATCT	165
**Gata-4**	CTGTCATCTCACTATGGGCA	CCAAGTCCGAGCAGGAATTT	259
**Hand2**	CCTTCAAGGCGGAGATCAAGA	CCTGTCCGGCCTTTGGTTTT	118
**Islet-1**	GCCTCAGTCCCAGAGTCATC	AGAGCCTGGTCCTCCTTCTG	308
**Mlc-2v**	GACCCAGATCCAGGAGTTCA	AATTGGACCTGGAGCCTCTT	163
**Nanog**	CACCCACCCATGCTAGTCTT	ACCCTCAAACTCCTGGTCCT	152
**Nkx2.5**	CAAGTGCTCTCCTGCTTTCC	GTCCAGCTCCACTGCCTTCT	130
**NPR-A**	CCAATTATGGCTCCCTGCTA	CGGTACAAGCTCCCACAAAT	198
**NPR-B**	TCATGACAGCCCATGGGAAA	GGTGACAATGCAGATGTTGG	209
**Oct-4**	GGATGCTGTGAGCCAAGG	GAACAAAATGATGAGTGACAGACAG	175
**PDGFRα**	GGGAAGGACTGGAAGCTTGGGGC	AGATGAGGCCCGGCCCTGTGAGG	154
**Sca-1**	TTTGAGACTTCTTGCCCATC	ACCCAGGATCTCCATACTTTC	159
**Sox2**	AAGGGTTCTTGCTGGGTTTT	AGACCACGAAAACGGTCTTG	150
**Tbx5**	GGAAAGATGAGGAATGTTCCAG	GTGTTACAGCTGATGTCCTCCA	223
**Tbx20**	CCCCGCTGCCAGCCAGGCTCTA	GTGCACCCGTGGCTGGTACTTATGC	167
**Tcf21**	GGCCAACGACAAGTACGAGA	GCTGTAGTTCCACACAAGCG	129
**Troponin T**	GCGGAAGAGTGGGAAGAGACA	CCACAGCTCCTTGGCCTTCT	127
**Vimentin**	GCCGAAAGCACCCTGCAGTCA	GCCTGCAGCTCCTGGATCTCTTCA	146
**vWF**	GATGCCCCAGTCAGCTCTAC	TCAGCCTCGGACAACATAGA	131
**Wnt1**	CACGGCACAGGGTATGAGAG	GTTGGGGCCACTCCAGATAC	128

**α-MHC**	AACCAGAGTTTGAGTGACAGAATG	ACTCCGTGCGGATGTCAA	130
**β-MHC**	ATGAGACGGTGGTGGGTTT	CTTTCTTTGCCTTGCCTTTG	117
**18S**	ACTTTTGGGGCCTTCGTGTC	GCCCAGAGACTCATTTCTTCTTG	96

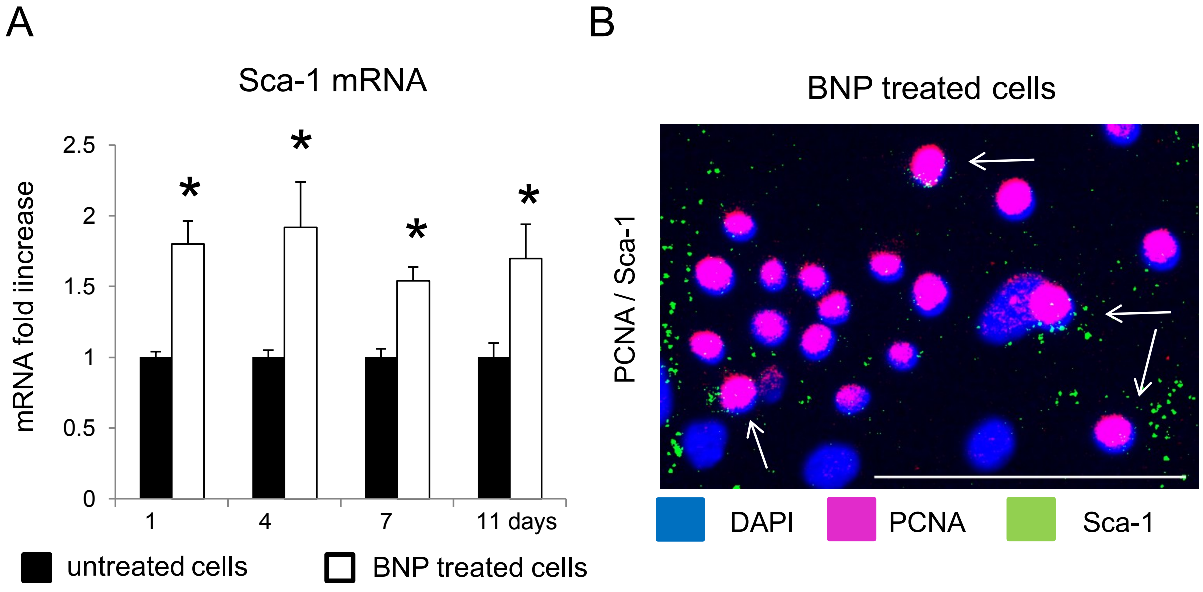

__Supplemental Figure 1.__ **BNP induces Sca-1⁺ cell proliferation. A.** Quantitative relative expression of mRNA coding for Sca-1 in untreated and BNP treated cells. n= at least 7 different experiments per group. **B.** Representative immunostaining of cells cultured 4 days with BNP and stained with antibodies against the Proliferating Cell Nuclear Antigen (PCNA) (pink), Sca-1 (green) and DAPI (blue). White arrows highlight proliferating Sca-1^{+} cells. Scale bar represents 100 μm.

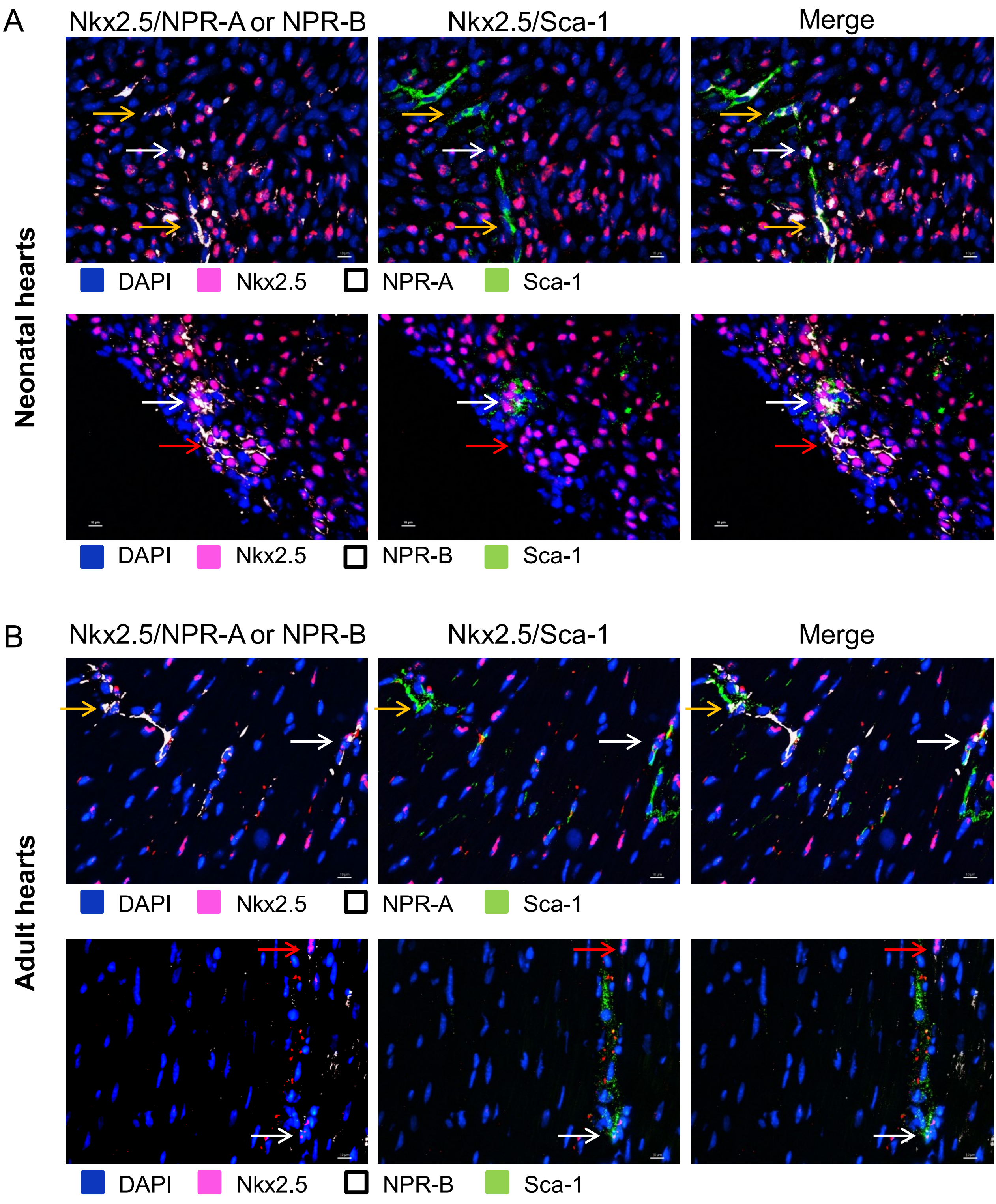

Supplemental Figure 2. NPR-A and NPR-B receptors are expressed on Nkx2.5$^+$ Sca-1$^+$ cells in neonatal and adult hearts. Immunostainings of neonatal (A) and adult murine hearts (B). Scale bars represented 10 μm. The yellow arrows point to Sca-1$^+$ Nkx2.5$^-$ cells expressing NPR-A or NPR-B, the white arrows point to Nkx2.5$^+$ Sca-1$^+$ cells and the red arrows point to Sca1$^-$ cells expressing Nkx2.5 and NPR-B.

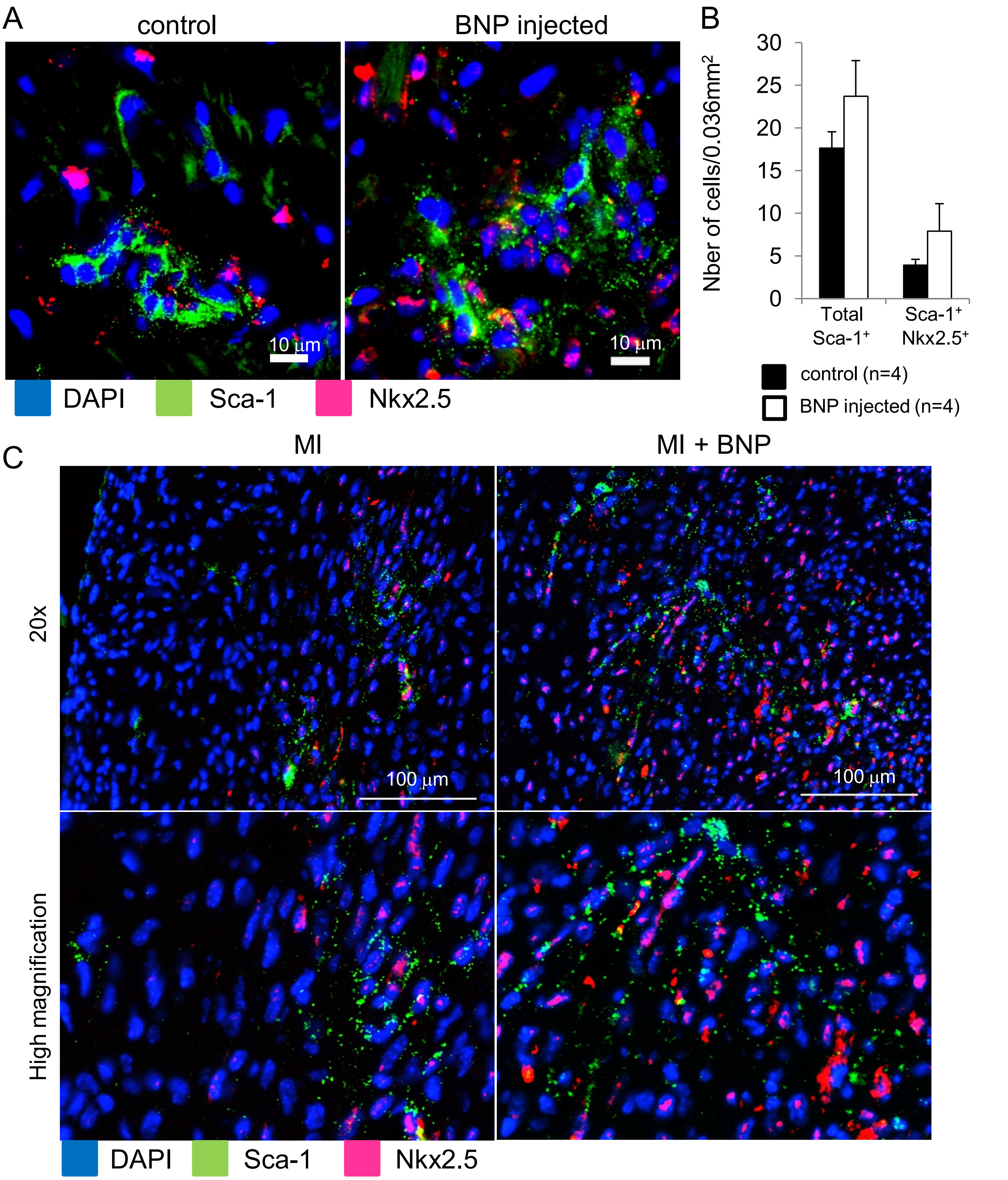

<u>Supplemental Figure 3.</u> BNP treatment in neonatal and infarcted hearts leads to increased number of Sca-1$^+$ Nkx2.5$^+$ cells. A. Heart sections from control or BNP injected neonatal mice. **B.** Quantification of the number of Sca-1$^+$ cells expressing Nkx2.5 in the hearts of control or BNP injected mice. Data were obtained from at least 6 different pictures per mouse, *n* = 4 mice per group. Each section covered an area of 0.036 mm^2. Data are means ± SEM. **C.** Heart sections, from infarcted mice injected or not with BNP. Representative pictures of x 20 and high magnification of the infarcted area of the infarcted hearts 10 days afer myocardial infarction (MI).

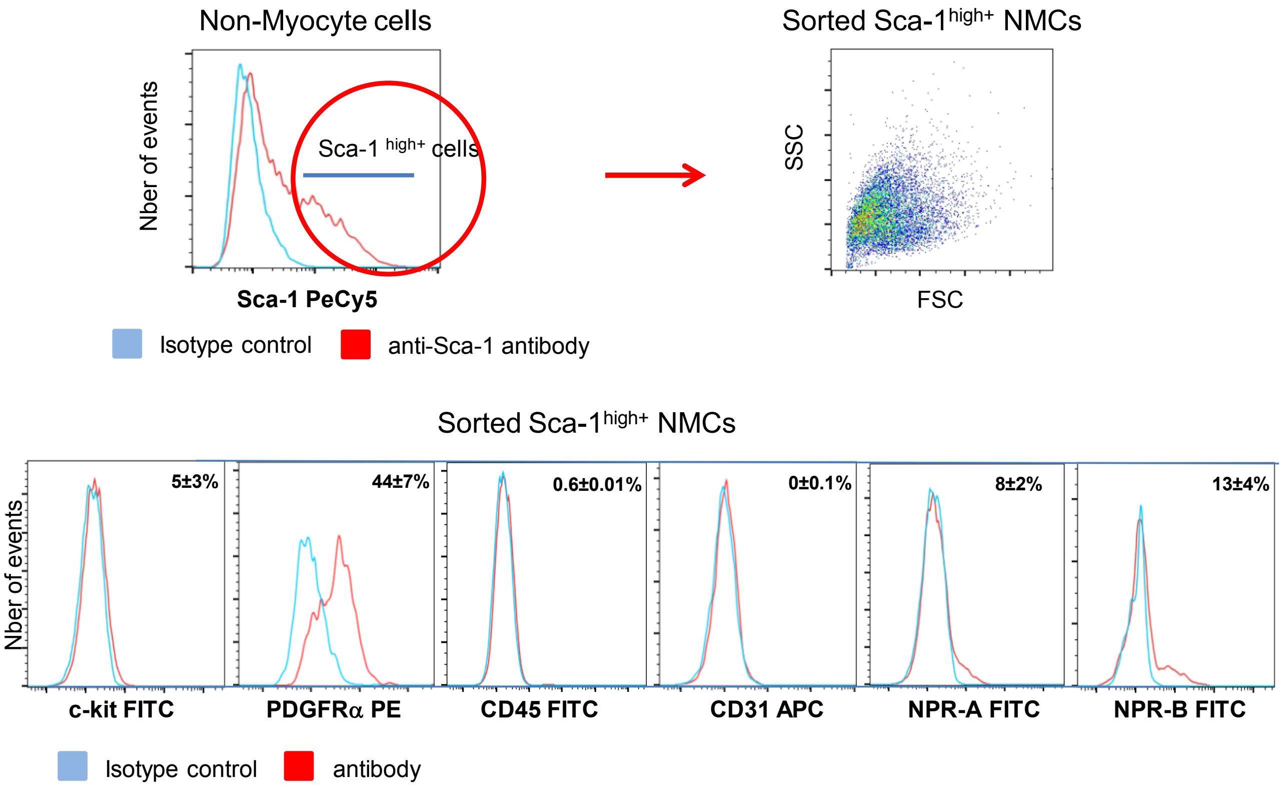

<u>Supplemental Figure 4.</u> Phenotypic characterisation of the sorted Sca-1^{high+} cells. Representative histograms and dot plot (right) of non myocyte cell (NMC) sorting for high Sca-1 expression. The histograms allow to characterize the Sca-1^{high+} cells for the expression of c-kit, PDGFRα, CD45, CD31 and NPR-A and NPR-B proteins. The numbers represent the percentage of the positive cells compared to the total number of Sca-1^{high+}cells and are the means ± SEM of 3-11 different experiments.

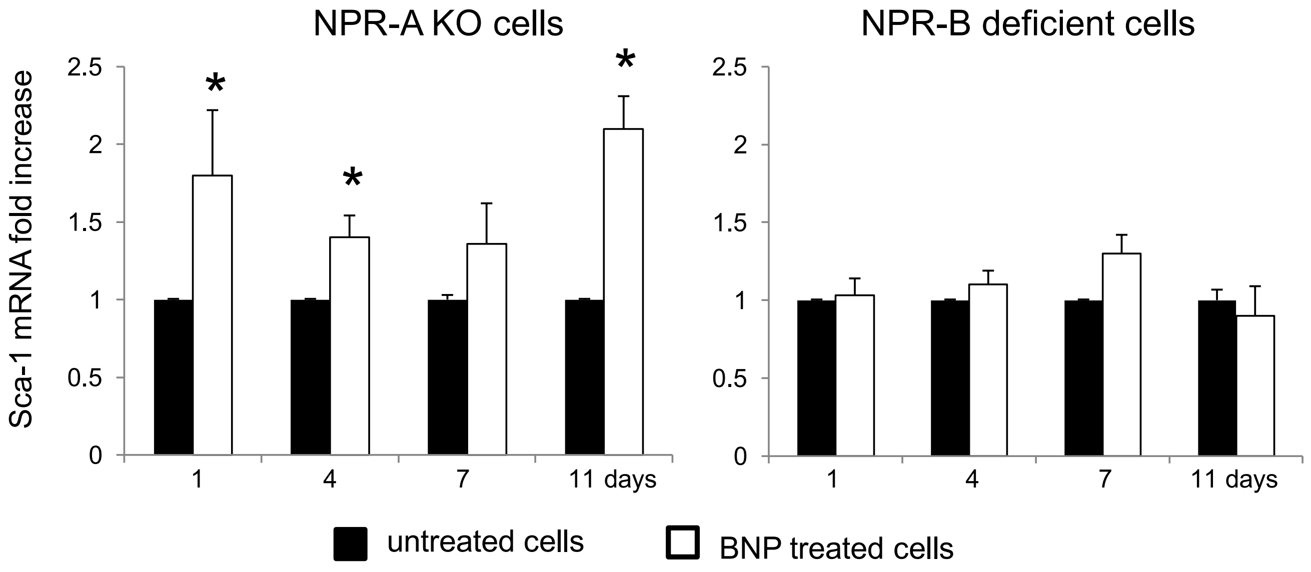

<u>Supplemental Figure 5.</u> BNP induced increased Sca-1 mRNA expression only in presence of NPR-B receptor. Quantitative relative expression of mRNA coding for Sca-1 in untreated and BNP treated NPR-A and NPR-B deficient NMCs (n= at least 6 different experiments at different time point). All the results are means ± SEM, * p<0.05 versus untreated cells.

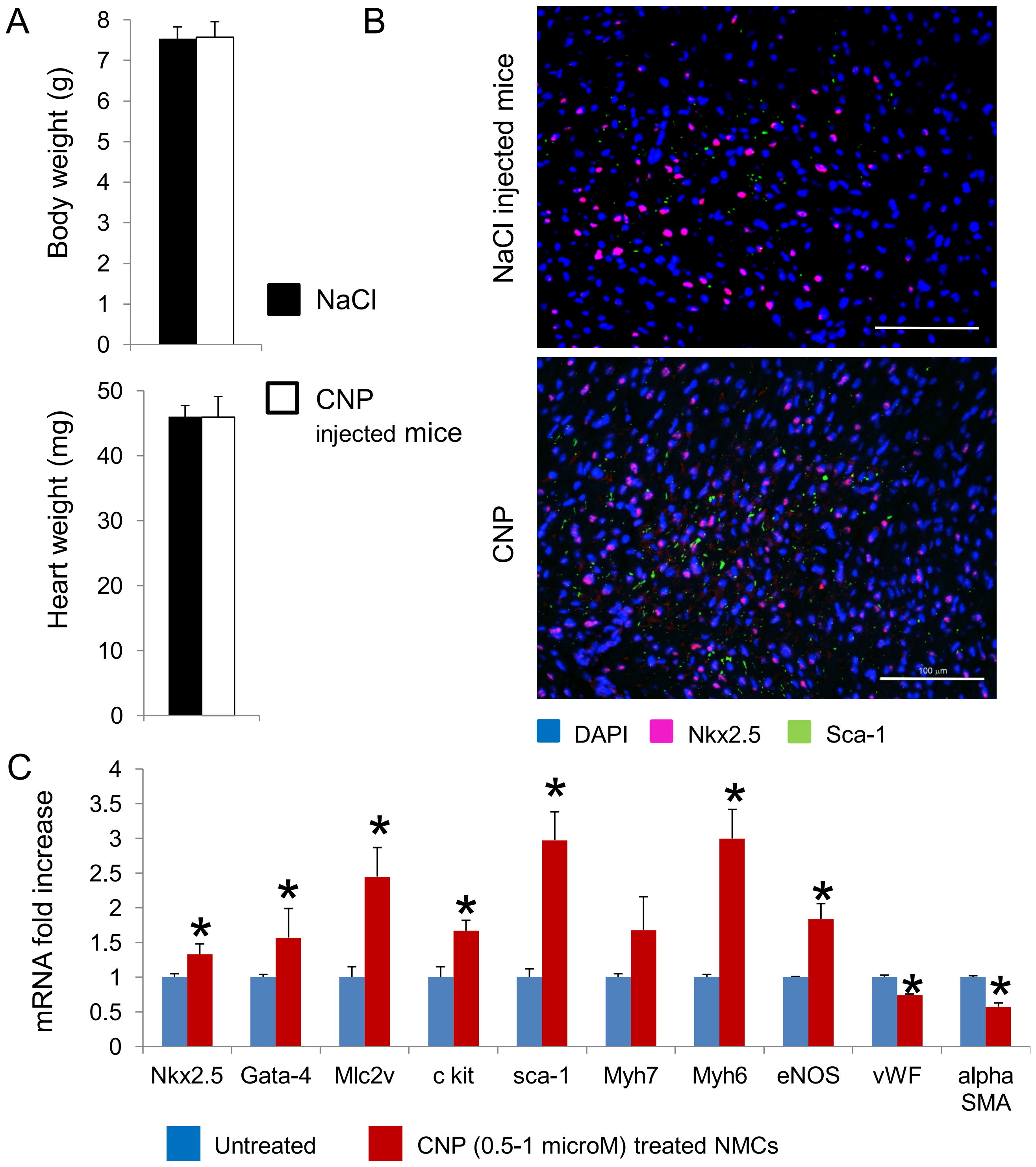

Supplementary Figure 6. CNP effect on cardiac Non Myocyte Cells *in vivo* and *in vitro*.
A-B. Newborn mice (4 days old) were injected with CNP intraperitoneally every 2 days for up to 2 weeks. Mice were sacrificed 2 days after the last injection. CNP treatment had no effect on body or heart weights. However the numbers of Sca-1$^+$ cells and of Sca-1$^+$ Nkx2.5$^+$ cells was increased (**B**). n=4 mice per group **C.** Non Myocyte cells were isolated from neonatal hearts and treated with CNP in differentiation medium for up to 3 weeks. In presence of CNP, NMCs upregulated cardiomyocyte specific genes (Nkx2.5, Gata-4, Mlc-2v, Myh6). n= 9 different experiments and * p< 0.05. vWF: von Willbrand Factor, SMA : smooth muscle actin.

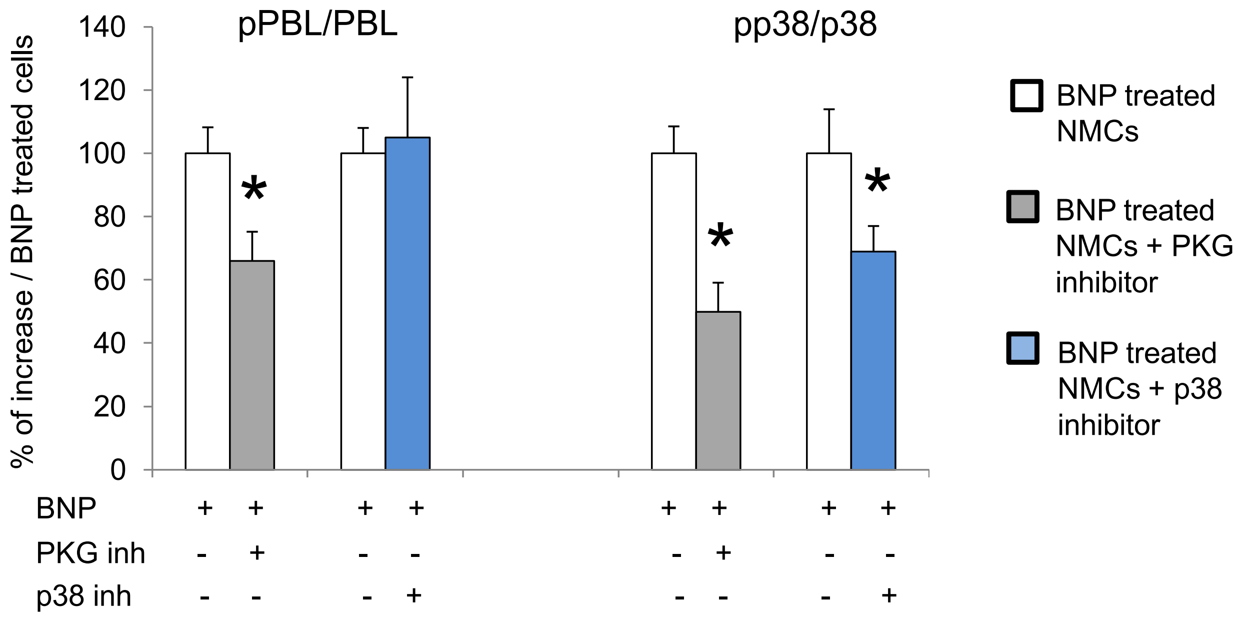

<u>Supplemental Figure 7.</u> BNP induced PKG activation, which is necessary for at least a part of p38 MAPK phosphorylation. NMCs were treated 15-30 min with BNP and with a PKG inhibitor or a p38 inhibitor. n= at least 6 different experiments per group. The data were related to the average of BNP treated NMCs. All the results are means ± SEM, * $p<0.05$ versus BNP treated cells.

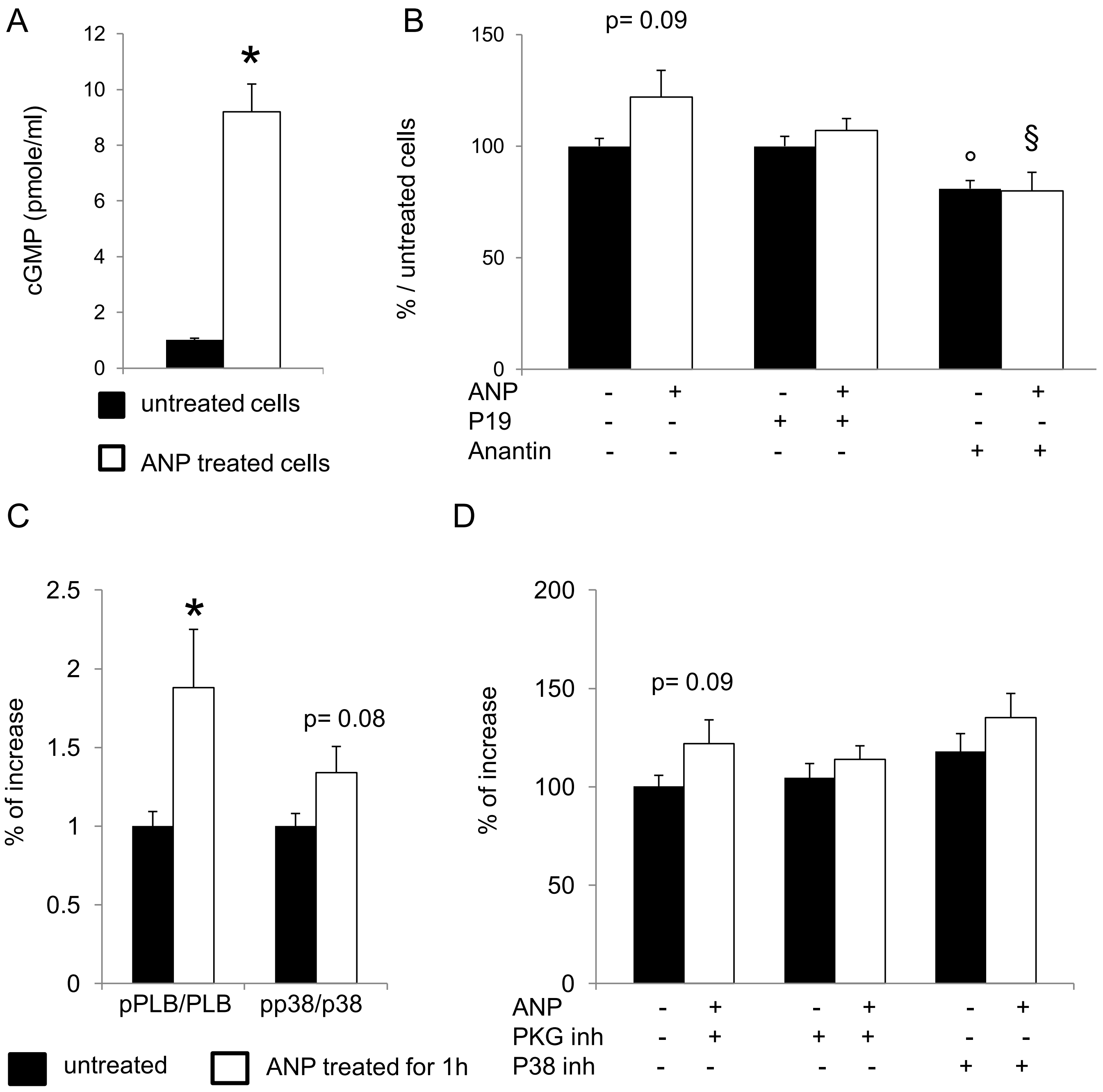

Supplemental Figure 8. ANP has a moderated effect on Sca-1+ cell proliferation. A. cGMP levels were measured in the non myocyte cell supernatants (n=4-7) 1h after ANP treatment (1μM). **B.** Sorted Sca-1^{high+} cells isolated from wild type hearts were cultured with ANP in presence or not of NPR-B receptor antagonist (P19, 0.5-1 microM) and of an ANF antagonist (Anantin, 0.2 microM). The number of cells was counted after 9-11 days of culture and the results were related to untreated cells. **B**. Quantification of the data from western blot analysis expressed relatively to the average of untreated cells. Sorted Sca-1^{high+} cells were or not treated 1h with ANP. **C**. Number of sorted Sca-1^{high+} cells after 11 days of culture with or without ANP and PKG or p38 inhibitor. The data were related to the average of untreated cells. **A-C**: n= at least 4 different experiments, All the results are means ± SEM, * p<0.05 versus untreated cells, ° p< 0.05 versus the untreated cells without inhibitors, § p< 0.05 versus the ANP treated cells without inhibitors.
